# A Pharmacokinetic and Metabolism Study of the TRPC6 Inhibitor SH045 in Mice by LC-MS/MS

**DOI:** 10.3390/ijms23073635

**Published:** 2022-03-26

**Authors:** Xiao-Ning Chai, Friedrich-Alexander Ludwig, Anne Müglitz, Yuanyuan Gong, Michael Schaefer, Ralf Regenthal, Ute Krügel

**Affiliations:** 1 Rudolf Boehm Institute for Pharmacology and Toxicology, Leipzig University, 04107 Leipzig, Germany; xiao-ning.chai@medizin.uni-leipzig.de (X.-N.C.); anne.mueglitz@medizin.uni-leipzig.de (A.M.); gong.yuanyuan@medizin.uni-leipzig.de (Y.G.); michael.schaefer@medizin.uni-leipzig.de (M.S.); 2 Department of Neuroradiopharmaceuticals, Institute of Radiopharmaceutical Cancer Research, Helmholtz-Zentrum Dresden-Rossendorf, 04318 Leipzig, Germany; f.ludwig@hzdr.de; 3 Clinical Pharmacology, Rudolf Boehm Institute for Pharmacology and Toxicology, Leipzig University, 04107 Leipzig, Germany; ralf.regenthal@medizin.uni-leipzig.de

**Keywords:** cytochrome P450 enzyme, kidney, larixol, LC-MS/MS, lung, mice, microsomes, pharmacokinetics, toxicity, SH045, TRPC6 inhibitor

## Abstract

TRPC6, the sixth member of the family of canonical transient receptor potential (TRP) channels, contributes to a variety of physiological processes and human pathologies. This study extends the knowledge on the newly developed TRPC6 blocker SH045 with respect to its main target organs beyond the description of plasma kinetics. According to the plasma concentration-time course in mice, SH045 is measurable up to 24 h after administration of 20 mg/kg BW (i.v.) and up to 6 h orally. The short plasma half-life and rather low oral bioavailability are contrasted by its reported high potency. Dosage limits were not worked out, but absence of safety concerns for 20 mg/kg BW supports further dose exploration. The disposition of SH045 is described. In particular, a high extravascular distribution, most prominent in lung, and a considerable renal elimination of SH045 were observed. SH045 is a substrate of CYP3A4 and CYP2A6. Hydroxylated and glucuronidated metabolites were identified under optimized LC-MS/MS conditions. The results guide a reasonable selection of dose and application route of SH045 for target-directed preclinical studies in vivo with one of the rare high potent and subtype-selective TRPC6 inhibitors available.

## 1. Introduction

Transient receptor potential (TRP) channels translate extracellular signals into dynamic alterations of intracellular cation concentrations, thereby triggering diverse cellular responses in health and disease. TRPC6 (sixth isoform of the canonical TRP channel subfamily) is closely related to TRPC3 and TRPC7. TRPC6 is a poorly selective cation channel with slightly higher Ca^2+^ permeability compared to Na^+^ influx. It mediates increases in intracellular Ca^2+^ and membrane depolarization [1], causing secondary effects that include coupling to signaling cascades that affect gene expression and cell proliferation [2]. TRPC6 channels are physiologically activated by the second messenger diacylglycerol (DAG) downstream of G_q/11_ protein-coupled receptors [3], receptor-mediated PLC_γ_-signaling [4,5]. Furthermore, recently identified Ca^2+^-binding sites in TRPC6 that sense intracellular concentrations qualify Ca^2+^ as an important regulator of channel activity [6]. In addition, TRPC6 are likely to be activated downstream of primary sensors of mechanical stimulation and to act as amplifier of cellular mechanosensory signaling cascades [7,8,9].

In line with the universal second messenger function of intracellular Ca^2+^, TRPC6 are widely expressed in numerous mammalian tissues, e.g., kidney, lung, heart, and brain [10], and contribute to specific cell functions like maintenance of slit-diaphragm structure and function of renal podocytes and mesangial cells, pulmonary endothelial permeability, smooth muscle contraction, and neuronal protection against ischemia [1,7].

Dysfunctions of TRPC6 either genetic or acquired with gain or with loss of function are associated with severe human pathologies. In particular, high expression of TRPC6 is observed in kidney, and mutations in the TRPC6-encoding gene are associated with progressive glomerular diseases like primary or secondary focal segmental glomerulosclerosis (FSGS), glomerulosclerosis as consequence of autoimmune glomerulonephritis or associated with diabetes mellitus type-1 or hypertension [11,12]. Though detailed TRPC6-related involvement remains to be evaluated, podocyte TRPC6 activation occurs via the angiotensin 1 receptor, and apart from increasing open-probability of already membrane-bound TRPC6 this induces their transfer to the cell surface [11]. TRPC6 channels interact with essential proteins in podocytes like podocin, nephrin, and others, all together regulating slit diaphragm filter function whose disruption allows proteins such as albumin, antithrombin, or the immunoglobulins to pass, and induces nephrotic syndrome.

In lung, another current subject in search for treatment options, increased TRPC6 responses of smooth muscle and vascular endothelial cells are associated with acute hypoxic vasoconstriction [13] but also with idiopathic pulmonary arterial hypertension (IPAH) [14] and lung ischemia-reperfusion edema (LIRE) [15,16]. 

As result of a natural compound strategy for new TRPC6-blocking chemical entities, congeners of the labdane diterpene (+)-larixol, a component from *Larix decidua* turpentine traditionally used for inhalation, larixyl monoacetate [17] and the more stable larixyl-6-carbamate [18] with propitious *IC*_50_ of about 0.5 µM in Ca^2+^ influx assays were identified. Promising, the latter turned out as a strong inhibitor of various FSGS-related TRPC6 channel mutants, including the highly active M132T and R175Q variants [18]. 

Further diversification of (+)-larixol led to the *N*-methylcarbamate congener SH045 generated in a cost-effective and straightforward semisynthetic two-step protocol from its abundant parent compound [19]. With an *IC*_50_ value of 5.8 nM SH045 favorably compares with the few small molecule inhibitors with high potencies and marked sub-type selectivity over TRPC3 and TRPC7 like BTDM [20] and the orally available SAR7334 [15] and BI 749,327 [19,21]. 

The promising inhibitory effect on gain-of-function of TRPC6 mutants of its precursor, advanced SH045 as a candidate for preclinical research in vivo. This was further supported by inhibition of 1-oleyl-2-acetyl-sn-glycerol (OAG)-induced Ca^2+^ entry through TRPC3/6 channels of rat pulmonary arterial smooth muscle cells (PASMC), the reduced edema formation after reperfusion of ischemic lungs explanted from mice, an ex vivo model of LIRE [19] and by its bioavailability after exploratory intraperitoneal (i.p.) administration in mice [22]. 

Recently, a methodological work on validation of high-performance liquid chromatography and coupled tandem mass spectrometry (LC-MS/MS) provided promising pilot data on the pharmacological profile of SH045 [22]. Using the basics of this analytical method, the present study provides requisite data on pharmacokinetics, biodistribution, metabolism, and tolerability after repeated administration to use SH045 for pharmacological inhibition of TRPC6 channels in disease models in vivo.

## 2. Results

### 2.1. LC-MS/MS Methods

A validated method for quantification of SH045 in mice plasma after optimized sample preparation based on LC-MS/MS was reported recently [22]. The method was developed further for quantification of SH045 in various tissues and urine from mice and in microsomal preparations. Details on resulting method adaptation and quality parameter requirements [22] are given in Table S1 of Supplementary Materials SR1. 

### 2.2. Pharmacokinetics, Disposition and Metabolism Study

#### 2.2.1. Concentration-Time Profile of SH045 in Plasma after i.v. and p.o. Administration

**Figure 1:**
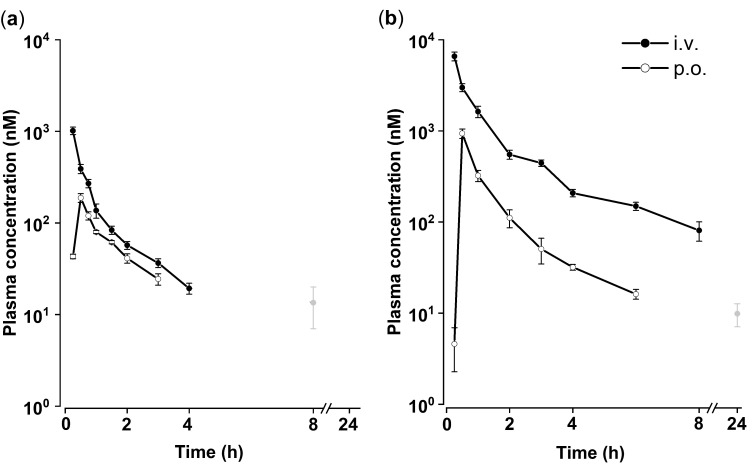
Mean plasma concentration-time profiles of SH045 after single intravenous (i.v.) and peroral administration (p.o.) of 2 mg/kg BW (**a**) and 20 mg/kg BW (**b**). Data are expressed as mean ± SEM (*n* = 6, independent values from 6 animals). Grey symbols represent means of only three determined plasma concentrations above the limit of quantification.

The concentration-time profile of SH045 (2 and 20 mg/kg body weight [BW]) in mouse plasma was recorded after single intravenous (i.v.) and peroral (p.o.) administration (Figure 1). Data were processed to calculate pharmacokinetic parameters by non-compartmental analysis (NCA) (Table 1). Peak concentration (*c_max_*) and peak time (*t_max_*) were directly obtained. The mean value for *t_max_* following i.v. administration was 0.25 h for 2 mg/kg BW and 20 mg/kg BW. Values of 0.50 h were observed for *t_max_* after both oral doses within a typical time frame for drug absorption. A 10-fold i.v. dosing of SH045 resulted in proportional 6.5- and 8.1-fold increases in *c_max_* and area under the curve extrapolated to infinity (*AUC_inf_*), respectively. Similarly, a 10-fold oral dosing resulted in 5.0- and 3.7-fold increases in *c_max_* and *AUC_inf_*, respectively. 

SH045 mean half-life (*t*_1/2_) varied in the range from 1.2 to 2.4 h. The nearly doubled half-life after the higher doses does not match linear first-order elimination kinetic characteristics as the values should be constant and independent of the dose and the route of administration. Likewise, different mean residence times (*MRT*) for SH045 were observed following intravenous doses (Table 1). 

Noteworthy, the analytical limit of quantification for SH045 (2 ng/mL, equal to 5.5 nM) is similar to the reported *IC*_50_-value of 5.8 nM in vitro, which is important to estimate SH045-mediated drug effects at later time points [19]. Here, at 24 h after 20 mg/kg BW (i.v.), values (grey points in Figure 1) indicate plasma concentrations still above the limit of quantification (*n* = 3 each). 

From *AUC_inf_* values (Table 1) oral bioavailability (*F*) for 2 and 20 mg/kg was calculated, which was low with 24.0% and 11.0%, respectively. The lower bioavailability following 10-fold p.o. dosing may be due to limited absorption or pre-systemic elimination. The percentage of extrapolated *AUC_extra_* as part of total *AUC_inf_* ranged from 3.7 to 5.0% (except 2 mg/kg p.o.), which meets the requirement that *AUC_0−t_* should cover at least 80% of *AUC_inf_* [23,24]. This indicates that NCA was well suited for sufficient evaluation of drug exposition. 

Calculated systemic clearances (*CL/F*) following both i.v. doses were similar and within biological variability (Table 1). With regard to oral bioavailability, values of total body clearance following oral administration were calculated as 95 mL/min/kg and 117 mL/min/kg after administration of 2 mg/kg and 20 mg/kg, respectively, corresponding to i.v. administration. Respective values calculated for volume of distribution (*Vz/F*) were 10.5 L/kg and 24.0 L/kg compatible with those after peroral administration indicating a widely unrestricted distribution throughout the whole body.

#### 2.2.2. Biodistribution and Tissue Binding of SH045 

To examine the applicability of SH045 in in vivo approaches, we investigated its tissue binding and biodistribution. Coincidentally to the high plasma protein binding [22], SH045 also extensively binds to kidney (98 %), liver (97%), and lung (97%) tissue (Supplementary Materials SR2, Table S2). 

**Figure 2:**
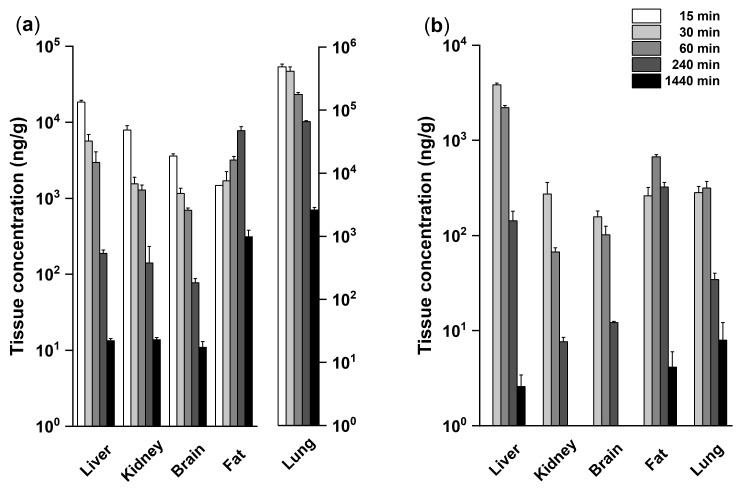
Time-dependent tissue concentration of SH045 in mice after single intravenous (i.v., **a**) and peroral (p.o., **b**) administration of 20 mg/kg BW. Note the higher SH045 concentration in lung (**a**, right panel) after i.v. administration. At some time points after oral administration tissue concentration was below the limit of quantification of 2 ng/mL. Data are expressed as mean ± SEM, *n* = 6.

Further we investigated the time-dependent distribution of SH045 in tissues (Figure 2). As expected by high the lipophilicity of SH045 and its calculated volume distribution, data indicate an almost complete systemic exposure to the drug, albeit considerably lower after oral administration, compatible with the limited oral bioavailability. 

In the parenchymatous organs liver, kidney, and brain SH045 (20 mg/kg, i.v.) attained similar effective drug concentrations over 24 h accompanied by a fast clearance from the organ. Of note, SH045 also entered the brain. 

After i.v. administration, fat tissue exhibited a prolonged offset of SH045, obviously related to high lipophilicity and low blood perfusion independent of administration route. 

A striking result is the extensive first-pass uptake of SH045 into the lung associated with a longer and higher exposition of pulmonary tissue compared to the other organs after i.v. administration, which was not found after oral administration. 

#### 2.2.3. Repeated Administration of SH045—Effects on Tissue Concentration and Histological Evaluation

In parallel to single i.v. administration of 20 mg/kg SH045 we performed a repeated administration five times once a day. As expected by the short *t*_1/2_, no accumulation of SH045 in plasma was observed 1 and 24 h after the last injection (not shown). Similarly, despite preferential extravascular appearance of SH045 no hints for drug accumulation in tissues were found (Table 2).

**Figure 3:**
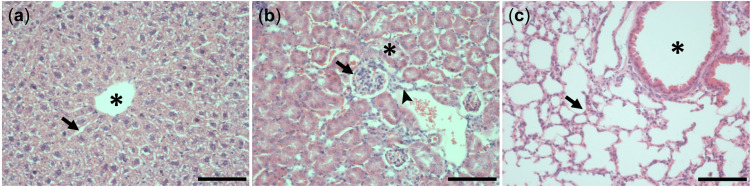
Examples of hematoxylin-eosin stained tissues slices of liver (**a**), kidney (**b**) and lung (**c**) of mice treated on five days, once a day, with SH045 (20 mg/kg BW, i.v.); (**a**): central vein (asterik), hepatic sinusoid (arrow); (**b**): renal corpuscle (arrow), proximal tubule (asterik), distal tubule (triangle); (**c**): alveoli (arrow), bronchiole (star). Scale bar: 100 µm.

Further, at hematoxylin-eosin stained tissues slices of mice liver, kidney and lung no histopathological changes were observed in the acute phase < 24 h (not shown) nor six days after single and repeated administration of SH045, respectively (Figure 3). In Supplementary Materials SR3 Figure S1 provides pictures of lower magnification compared to respective control slides with explanations.

#### 2.2.4. Appearance of SH045 in Urine 

Mouse renal elimination of SH045 was exploratorily monitored up to their first spontaneous micturition after i.v. administration (20 mg/kg BW). Samples of released urine were assigned to micturition periods of 10–30, 60–80, or 100–120 min following application. Mean urine concentrations of unmetabolized SH045 of 748 ± 31 (*n* = 3), 1056 ± 104 (*n* = 3), and 162 ± 17 nM (*n* = 6), respectively, were recorded. 

As no true urine volumes per time period were collected, a ratio of parent compound in urine related to the applied dose could not be determined. However, obtained values timely relate to corresponding drug plasma concentrations of 3035, 1655, and 560 nM SH045 after dosing. 

This observation reflects not only a timely renal elimination of the parent drug from systemic circulation, but also that an apparent amount of pharmacological active SH045 passes the kidney filter apparatus beyond 2 h after administration.

### 2.3. Studies on Hepatic Metabolism of SH045 In Vitro

Physiologically the metabolic fate of drugs is mainly affected by the activity of hepatic sinusoidal and canalicular transporters and conversion by hepatic cytochrome P450 enzymes [25]. 

Liver microsomes serve as an appropriate model for hepatic metabolism in vivo, which might address the general interest in species differences including possible pathways in human, too. With the assumption that phase I metabolism is the major pathway for drug elimination also in vivo [26], microsomes were used to determine kinetic parameters, which allowed an in vitro-in vivo extrapolation for clearance of SH045.

#### 2.3.1. Metabolism of SH045 in Mouse (MLM) and Human (HLM) Liver Microsomes In Vitro 

**Figure 4:**
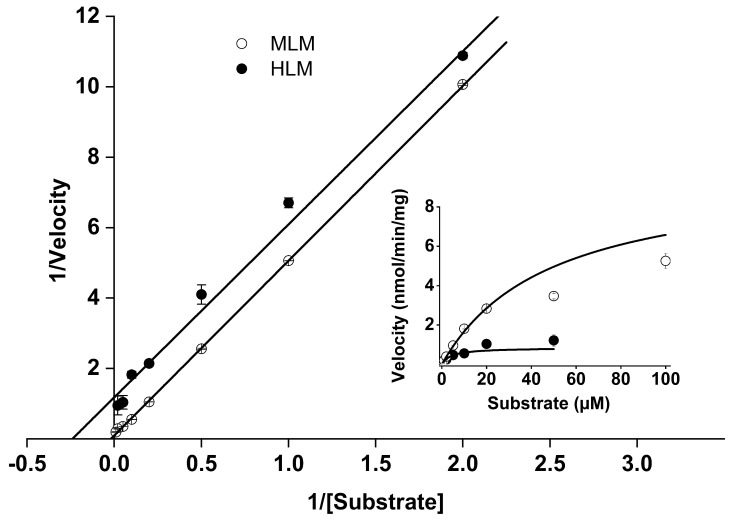
Metabolization rate of SH045 in mouse (MLM) and human (HLM) liver microsomes (0.5 mg/mL protein) in the presence of NADPH in relation to various substrate concentrations (SH045 0.5 to 100 µM) presented as Lineweaver-Burk-Plot. Inset: original data of Michaelis-Menten kinetics; velocity (nmol/min/mg) = ∆substrate concentration/incubation time/enzyme concentration. Data are given as mean ± SEM, *n* = 3.

Initially, the rate of NADPH-dependent microsomal conversion of SH045 was determined in MLM and HLM at different concentrations of SH045 (Figure 4) and, according to Michaelis-Menten kinetics and subsequent Lineweaver-Burk plot, *v_max_* (nmol/min/mg), and *K_m_* (µM) were computed for MLM: 9.8 ± 0.8 and 48.4 ± 3.9 and for HLM: 0.8 ± 0.1 and 4.1 ± 0.8, respectively.

On that basis, an appropriate concentration of 2 µM of SH045 was used in further in vitro investigations.

#### 2.3.2. Time-Dependent Metabolization of SH045 in MLM and HLM

**Figure 5:**
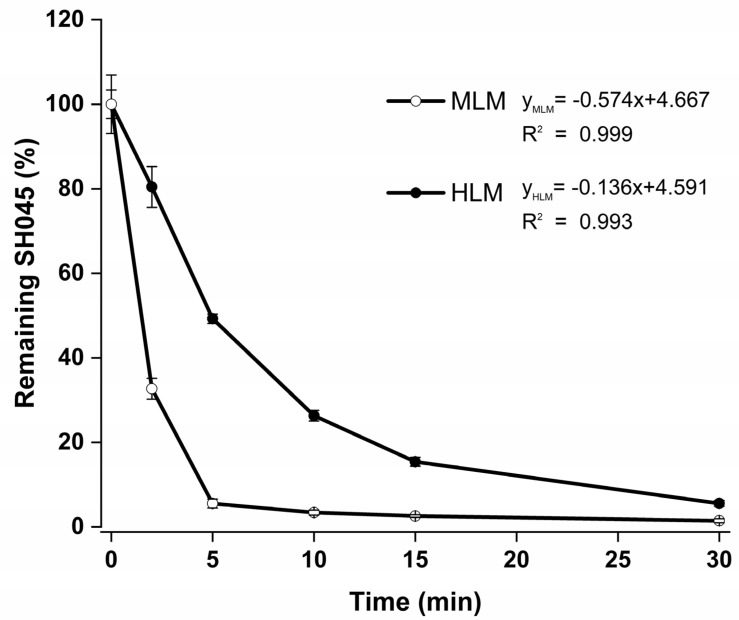
Time-dependent metabolization (“stability”) of SH045 by mouse (MLM) and human (HLM) liver microsomes (0.5 mg/mL protein each). The equation represents the calculation of in vitro half-life *t*_1/2_ from plotting of natural logarithm of remaining SH045 (%) versus time (*t*_1/2_ = *ln2/slope*) and the corresponding regression analysis for the linear range (not shown). Data are given as mean ± SEM, *n* = 3.

Examination of time-dependent metabolization (“stability”) of SH045 during incubation with species-specific microsomes in presence of NADPH revealed different velocities in MLM and HLM (Figure 5). The determined *t*_1/2_, amounting to 1.18 min for MLM and 4.85 min for HLM, respectively, was used for calculation of hepatic clearance *CL_hep_* parameters (Table 3). 

#### 2.3.3. Calculation of Hepatic Clearance Using In Vitro–In Vivo Extrapolation (IVIVE)

As hepatic clearance *CL_hep_* is a main determinant, which influences oral bioavailability, steady-state plateau concentration and half-life of a pharmacological active parent drug, the rate at which SH045 is metabolized by mouse and human liver and eliminated from the body thereafter can be predicted. 

IVIVE allows the use of the calculated intrinsic clearance *CL_int_*, equal to maximum activity of liver, from the microsomal metabolization (Figure 5, Table 3) to extrapolate the pharmacokinetic impact in vivo. 

In the widely used concept of the “well-stirred” model [28], liver represents a single uniform compartment. It is further assumed that the concentration of unbound SH045 leaving the liver is in equilibrium with its unbound concentration in hepatocytes (detailed calculation in Supplementary Materials SM8, summarized values in SR4). Briefly, microsomal intrinsic clearance was converted to hepatic clearance: *CL_hep_* = *Q* × *f_u_* × *CL_in_*_t_/*Q* + (*f_u_ × CL_int_*), where *f_u_* is the unbound fraction of SH045 in blood and *Q* is the hepatic blood flow in mice [29].

Considering (*i*) calculated *CL_int_* value from MLM (Table 3), (*ii*) the estimated unbound SH045 fraction of 0.3% [22] and (*iii*) published *Q* of 90 mL/min/kg [30], the hepatic clearance of SH045 in mice was translated to 11.95 mL/min/kg.

This is equal to only a minor hepatic amount of about 8.3% and 11.4% of total plasma clearance calculated after i.v. administration of 2 and 20 mg/kg SH045. This means in effect a predominant extrahepatic clearance of SH045. 

### 2.4. Identification of Metabolic Pathways of SH045 and Structural Elucidation of Metabolites

To characterize the biological fate of a so far unknown compound it was investigated whether metabolism is part of systemic clearance. Therefore, experiments on cytochrome P450 isoenzyme inhibition were performed with potent isoform-specific inhibitors in liver microsomes. 

**Figure 6:**
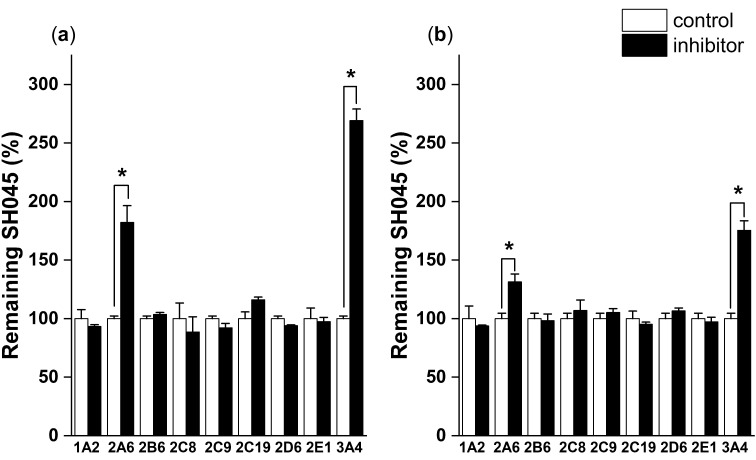
Investigation of phase I metabolism pathways for SH045 by cytochrome P450 isoenzyme inhibition in mouse (**a**) and human liver microsomes (**b**). The remaining SH045 in the incubation assay with inhibitor is presented as percentage of control without inhibitor normalized to 100%. CYP-isoform specific inhibitors: CYP1A2: ciprofloxacin, CYP2A6: pilocarpine, CYP2B6: ticlopidine, CYP2C8: quercetin, CYP2C9: sulfaphenazole, CYP2C19: fluvoxamine, CYP2D6: quinidine, CYP2E1: 4-methyl pyrazole, CYP3A4: ketoconazole. Significant differences were analyzed using raw data. Data are mean ± SEM, *n* = 3, * significant with *p* < 0.05.

SH045 is mainly metabolized by two cytochrome P450 isoenzymes, CYP3A4 and CYP2A6, both providing various metabolites mainly by oxidation reactions, which at least in part might be pharmacologically active as well (Figure 6). 

These and further endogenously conjugated phase II derivatives are likely to be more soluble and therefore should occur finally in measurable amounts in urine. The attempt to identify expected metabolites in urine was strengthened by accompanying investigations using microsome fractions and LC-MS/MS [22,25,31,32]. 

**Figure 7:**
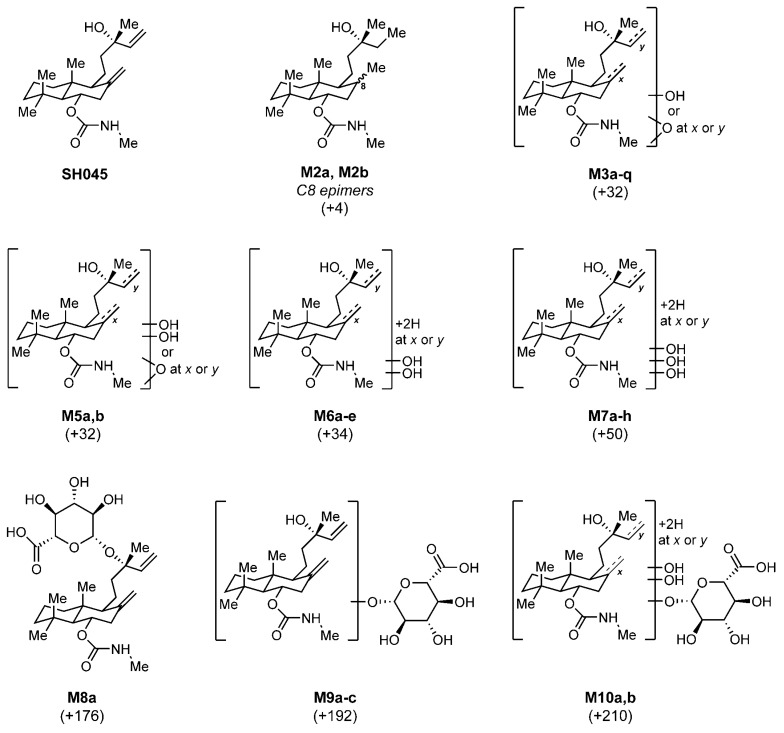
Structure of SH045 and proposed metabolites detected in MLM, HLM (for both: NADPH, UDPGA) and urine according to LC-MS/MS data. Mass shifts in relation to SH045 in brackets. For detailed information about occurrence of metabolites see Table 4. For discussion of EPI (MS/MS) data and more detailed assignment of selected structures see Supplementary Materials SR6.

Thereby, in mouse urine 17 metabolites of SH045 were detected, far less than the 34 metabolites found in microsomal incubations in total, in the majority hydroxylated, but also glucuronidated congeners [33]. All detected metabolites and observed biotransformations are summarized in Table 4 and Figure 7. More detailed descriptions of metabolites identified, relevant MRM chromatograms, EPI spectra, and interpretation of fragmentation patterns are given in the Supplementary Materials SR5 and Figures S2–S4.

Taken together, metabolites found and proposed reactions behind are consistent with the participation of cytochrome P450 isoenzymes 3A4 and 2A6 in the metabolism of SH045. 

## 3. Discussion and Conclusions

The present study extends the knowledge on the newly developed TRPC6 blocker SH045 with respect to its main target organs beyond the description of plasma kinetics.

According to the plasma concentration-time course in mice, SH045 is measurable up to 24 h after administration of 20 mg/kg BW (i.v.) and up to 6 h orally. The plasma half-life was short and oral bioavailability was rather low, but contrasted by high potency of SH045. However, in the present study dosage limits were not worked out and the absence of safety concerns for 20 mg/kg BW supports further dose exploration. First time, the disposition of SH045 as a highly potent TRPC6 blocker is described. In particular, a high extravascular distribution, most prominent in lung, was observed and moreover, SH045 appeared in considerable concentrations in urine. A high portion of extrahepatic elimination is concluded.

Present data confirmed a rapid absorption of SH045 into the vascular system followed by a large systemic distribution with high organ load, possibly driven by interstitial diffusion and perfusion mediated transport, recorded as fast elimination from plasma and strongly reflected by high volumes of distribution (*V_z_*) [22]. 

SH045 strongly distributes in target tissues, including lung, kidney, and also liver. While tissue binding to parenchymatous organs *per se* does not inform about ratio of tissue penetration, it contributes to resulting plasma concentration-time profiles. Higher tissue concentrations may prolong target effects by the pharmacological active fraction, released from the depot of unspecific binding sites within equilibrium between bound and unbound ratio. 

A striking result is the extensive first-pass uptake of SH045 into the lung to about the 20-fold of respective plasma concentration. This was associated with longer and higher exposition of pulmonary tissue compared to the other organs after i.v. administration, but was not found to this extent after oral administration [34]. Such high pulmonary accumulation associated with low back diffusion, but remaining high local drug concentration is also known for other drugs [35], which will inevitably result in a lower disposition of drug that enters the systemic circulation. In addition, for SH045 as a diterpene congener, clearance by lung cannot be ruled out [36,37].

Not least, strongly expressed TRPC6 channels and high lasting concentrations of SH045 in the lung may correlate with traditional use of *Larix decidua* resins against breathing problems [38,39].

In opposite to parenchymatous organs, visceral white fat is also a remarkable drug reservoir with delayed *c_max_* and prolonged back diffusion characteristic for lipophilic compounds in poorly perfused organs. This might be of interest with respect to the discussed involvement of TRPC6 in adipocyte differentiation [40].

Despite wide tissue distribution, no accumulation in plasma or organs, neither lung nor any acute tissue toxicity were observed after repeated administration. 

The short plasma half-life implies that repeated administration may be needed to acquire steady-state plasma levels. However, it is worth noting that plasma and tissue concentrations measured near the analytical limit of quantification at later time points are similar to the reported *IC*_50_ value in vitro, essential for SH045-mediated drug effects. Higher doses were not explored here, but may be suitable and tolerable, too. 

In view of SH045-mediated effects as TRPC6 blocker in animal models of renal pathology, the unmetabolized SH045 appeared in urine in considerable concentrations such as 160 nM after 2 h. Of note, this corresponds to about 30-fold of *IC*_50_ reported for SH045 in vitro. This is of particular importance as TRPC6 is described to be expressed in podocytes located in the bowman’s capsule on the glomerular basement membrane directed to the urinary filtrate [41].

SH045 plasma kinetics showed a clear dose-proportional increase in exposition (*AUC_inf_*) regardless of administration route. The slightly lower increase of *AUC_inf_* after the 10-fold oral dose is unclear. A limited gastrointestinal absorption from the drug formula used may be involved and may be subject of further optimization. It cannot be ruled out that SH045 becomes a substrate of outward-directed ABC cassette transporters like p-glycoprotein at the apical intestinal membrane at higher oral doses. In general, an extrahepatic clearance by intestinal or gut microbiota biotransformation is also conceivable. For SH045 a difference in terminal half-life was observed among routes of administration. This somewhat unexpected result may be caused as not enough samples with measurable concentrations at later time points at the used dose levels could be obtained and therefore (re)distribution processes, which may impact the terminal half-life, could not be considered. 

A simulated recalculation including the late low concentration values into the NCA generates a reduced *AUC_extra_* percentage from 4.2 to 0.8%, however the drug exposition calculated by current NCA remains unaffected. Further, this modeling revealed that the half-life of the terminal phase determined by drug elimination may be prolonged up to a range of 4 to 5 h. The issue of SH045 half-life should be addressed in future studies. Higher dose levels (than the exploratory once used) and a higher data density at later time points may overcome this uncertainty.

The knowledge on biotransformation of new potential drugs is mandatory for safety and efficacy evaluation and part of preclinical studies.

Apart from the marked amount of parent compound in urine, the metabolic fate of SH045 is determined by hepatic cytochrome P450 isoenzymes 3A4 and 2A6. The monooxygenase CYP3A4 decisively contributes to low oral bioavailability by hepatic, less probably, but possible also by pulmonary and intestinal metabolization of SH045. 

The aim for distinct detection of such metabolites in biological samples was met by application of four different gradient elution methods for LC-MS/MS. For all of them a short run-time enabled measurements using different MS scan modes within a reasonable period. Besides unchanged SH045, several metabolites appeared in urine in line with cytochrome P450 isoenzymes mainly identified as hydroxylation reactions but also as phase II glucuronidation. This may provide information about potential structurally similar metabolites, in addition to chemical structure modifications of the parent, as future subjects for drug optimization.

Regardless of the described manifold metabolic conversions, in quantitative aspects the hepatic biotransformation does not represent the dominant elimination process and makes dose enhancement plausible.

Overall, the presented results from the in vivo and in vitro characterization of SH045 may guide a reasonable selection of dose and route of application. Thereby, the stage for target-directed future preclinical studies in vivo with one of the rare high potent and subtype-selective TRPC6 inhibitors available is prepared. 

## 4. Material and Methods 

### 4.1. Materials 

Larixyl-6-N-methylcarbamate ((1S,4S,4aR,8aS)-4-((S)-3-Hydroxy-3-methylpent-4-en-1-yl)-4a,8,8-trimethyl-3-methylenedecahydronaphthalen-1-yl methylcarbamate, SH045, TRPC6 inhibitor) was synthesized as described by Häfner et al. [19]. (+)-Larixol (internal standard, IS) was isolated from Larix decidua turpentine [17]. Liver microsomes from mice (CD-1, male) and human (pooled: 50 donors) were obtained from Fisher Scientific (Gibco, protein concentration: 20 mg/mL respectively). Further materials are provided in Supplementary Materials SM1. 

### 4.2. HPLC and Tandem Mass Spectrometric Methods (LC-MS/MS)

All analyses were performed on an Agilent 1260 Infinity quaternary HPLC system (Agilent Technologies, Waldbronn, Germany) coupled to a tandem QTRAP 5500 hybrid linear ion-trap triple quadrupole mass spectrometer (AB SCIEX, Concord, ON, Canada), operated in positive electrospray ionization mode. Data were registered and processed using Analyst software (Version 1.7.1, AB SCIEX). Multiquant software (Version 2.1.1, AB SCIEX) was used for linear regressions and calculations of SH045 concentrations. Identification of metabolites was performed by means of LightSight software (Version 2.3.0.152038, AB SCIEX). 

### 4.3. Quantification of SH045

Analysis of SH045 in samples from plasma, tissue, urine, and microsomal incubations was performed as previously described [22]. Details are described in Supplementary Materials SM2. 

### 4.4. Preparation of Standard Stock Solutions, Calibration and QC Samples, and Working Solutions for Metabolism Studies

Analytic stock solutions of SH045 and IS (1 mg/mL each) were prepared in ACN and further diluted to working solutions and calibration and QC samples as described recently [22]. 

### 4.5. Animals, Drug Administration and Sample Collection

Male C57BL/6 mice (25–30 g BW, Charles River, Sulzfeld, Germany) housed in groups under standard conditions and allowed access to lab chow and water ad libitum were used. The in vivo experiments were performed according to the national regulations of animal welfare and were ethically approved by the local committee on animal welfare and the local authorities. Animal experiments were performed under strict consideration of the 3R principles, fully complying with the ARRIVE guidelines [42]. For single SH045 administration mice were assigned to four groups of 12 animals each: 2 and 20 mg/kg BW applied via the i.v. and the p.o. route, respectively. Due to the recommended limited volume for blood collection, each mouse was assigned to a maximum of five different time points each out of 8 (for p.o.) and 9 (for i.v.) for blood collection resulting in six independent plasma concentration values per time point. 

SH045 was freshly prepared with DMSO (final concentration 0.5%) and Kolliphor^®^ EL (5% in water). At 7:00 a.m., one hour before drug administration, food was removed. For oral gavage and for i.v. administration into the tail vein the respective dose was applied in a volume of 3 mL/kg BW. 

Via heparin-coated catheters a maximum of 50 µL blood was collected from the tail vein at 0.25, 0.5, 1, 2, 3, 4, 6, 8, or 24 h after administration and immediately centrifuged at 4000× *g* at 4 °C for 10 min. For repeated administration 20 mg/kg BW SH045 were administered at 8:00 a.m. on five consecutive days, once a day. Blood was taken 1 h and 24 h after drug administration. Tissue samples were harvested from isoflurane-euthanized animals at indicated time points. Mice were transcardially perfused with PBS and dissected tissues were washed to remove remaining blood. Plasma and tissue samples were stored at −80 °C until analysis. Urine was collected by bladder puncture at indicated time points. 

### 4.6. Histopathological Examination of Tissue Repeatedly Exposed to SH045

Liver, kidney, and lung were collected after repeated administration, fixed and processed for inspection for histopathological alterations (Supplementary Materials SM3). 

### 4.7. Preparation of Samples for Analysis of SH045 and Its Metabolites by LC-MS/MS

For tissue analysis, homogenates (10% in phosphate-buffered saline, PBS) were prepared. Homogenate, plasma, and urine were mixed with IS and extracted with ice-cold ACN, centrifuged, filtered and stored in the autosampler at 6 °C until analysis in duplicates by LC-MS/MS (details in Supplementary Materials SM4). 

### 4.8. Determination of Kinetic Parameters of SH045 in Mice

Pharmacokinetics parameters for SH045 were determined based on non-compartmental model analysis (NCA) using the Phoenix WinNonlin Software 8.3 (Certara, Princeton, NJ, USA). The slope of the terminal phase of the SH045 concentration-time profile (*λz*) was estimated as absolute values from ≥3 data points using a log-linear regression analysis. The area under the concentration-time curve (*AUC*) for the time (*t*) providing the final measurable concentration (*AUC_last_*) was calculated using the mixed log-linear trapezoidal rule (linear up, log down). The *AUC_inf_* was computed as *AUC_last_* + *C_last_/λz*, where the estimated value of the latest data point is used. The systemic total body clearance from plasma (*CL_p_*) was calculated as applied dose divided by *AUC_inf_*. *MRT* was calculated as *AUMC_inf_/AUC_inf_*, where area under the first moment curve (*AUMC*) was defined as area under the curve of the product of time and the plasma drug concentration versus time from zero to infinity. Finally, the *AUC_extra_* extrapolates the *AUC* from the last measured time point to infinity. *Vz*, the apparent volume of distribution during terminal elimination phase was calculated by *Vz* = *Dose/AUC_inf_ × λz*, where *λz* is the plasma elimination rate constant. The apparent volume of distribution during the terminal phase following extravascular administration based on the fraction of dose absorbed *Vz/F* = *Dose*/(*AUC_inf_ × λz*). The bioavailability (*F*) of two oral doses SH045 doses were calculated with reference to identical doses administered by intravenous bolus application of SH045 as follows: *F* = *AUC_oral_*/*AUC_i.v._* Tissue binding of SH045 was analyzed exemplarily in lung, liver and kidney homogenate using ultrafiltration [43]. The concentration in the filtrate refers to the unbound SH045 concentration according to *tissue binding* (%) = (*c_tot_* − *c_filtrate_*)/*c_total_* × 100. For details see Supplementary Materials SM5.

### 4.9. Metabolization Studies in MLM and HLM and Calculation of Hepatic Clearance

For determination of *v_max_* and Michaelis-Menten constant *K_m_* of the NADPH-dependent microsomal depletion of SH045, incubations in MLM and HLM were performed. Testosterone instead of SH045 was used as positive control (Supplementary Materials SM6). 

Negative controls were obtained by omission of respective components. The overall velocity (*v*) of degradation was calculated from altered SH045 concentration (∆*c*) obtained from reduction peak area ratio of SH045 as: *v* = ∆*c/incubation time × concentration of enzyme* (nmol/min × mg). Mean maximum rate *v_max_* and *K_m_* were calculated using Lineweaver-Burk transformation. From *K_m_* an appropriate drug concentration of 2 µM was derived for in vitro determination of *t*_1/2_ required for further exploration of clearance. 

For this purpose, remaining concentrations of SH045 in microsomes were determined to calculate the intrinsic microsomal clearance *CL_int,micr_*, the intrinsic clearance *CL_int_*, equal to the maximum enzyme activity of liver and for in vitro-in vivo exploration of hepatic clearance *CL_hep_* in mouse and human using the well-stirred model [28]. The algorithm for these calculations is given in detail in the Supplementary Materials SM8.

### 4.10. Identification of Metabolization Pathways and Metabolites of SH045 in MLM and HLM

To determine possible metabolizing pathways of SH045 in MLM and HLM, an in vitro inhibition approach was performed with CYP-isoform specific inhibitors. 

To explore metabolites of SH045 formed by microsomes, DPBS, alamethicin (25 µg/mL) [44] and MLM or HLM (1.0 mg/mL) were pre-incubated [45], further incubated with SH045 MgCl_2_, NADPH and UDPGA, terminated, and processed for LC-MS/MS analysis as described. Positive controls with testosterone and 4-nitrophenol and negative controls were analyzed too (Supplementary Materials SM7).

### 4.11. Calculations and Statistical Analysis

Analyst Software (Version 1.7.1, ABSCIEX) was used to collect data and for further quantification with MultiQuantTM Software (Version 2.1.1, ABSCIEX). All data are presented as mean ± standard error of mean (SEM), except where otherwise specified. OriginPro 2017G (OriginLab, Northampton, MA, USA) was used for graphical representations.

## Figures and Tables

**Table 1:** Non-compartment analysis (NCA) of pharmacokinetic parameters in plasma following single intravenous and peroral administration of SH045 (2.0 and 20 mg/kg BW) in mice. Mean plasma concentration data presented in Figure 1 were used *.

Dose/ Route	*t_max_* (h)	*c_max_* (ng/mL)	*t*_1/2_ (h)	*AUC_0-t_* (ng × h/mL)	*AUC_extra_* (%)	*AUC_inf_* (ng × h/mL)	*MRT* (h)	*V_z_/F* (L/kg)	*CL/F* (mL/min/kg)	*F*
2.0 mg/kg i.v.	0.25	368.7	1.28	338	3.7	351.2	0.75	10.5	94.9	1.0
20 mg/kg i.v.	0.25	2390.1	2.37	2724	4.2	2844.1	1.52	24.0	117.2	1.0
2.0 mg/kg p.o.	0.50	68.3	1.15	69	17.5	84.2	1.78	39.5	395.8	0.24
20 mg/kg p.o.	0.50	340.6	1.86	298	5.0	314.2	1.68	170.6	1060.7	0.11

* except of data points marked in grey color in Figure 1.

**Table 2:** Tissue distribution of SH045 at 24 h after single (1×) and repeated (5 days, once a day) i.v. administration of SH045 (20 mg/kg BW). Data are expressed as the mean ± SEM, *n* = 6.

SH045 20 mg/kg; i.v.	Concentration (ng/g)				
	Liver	Kidney	Brain	Fat	Lung
1×	14.2 ± 5.2	14.8 ± 5.9	14.8 ± 7.6	333.0 ± 61.2	2167 ± 414
5×	17.5 ± 4.6	17.8 ± 4.4	19.6 ± 5.5	369.2 ± 50.0	2390 ± 1019

**Table 3:** Liver microsomal stability and clearance of SH045 in vitro and its calculated hepatic clearance in vivo.

Microsomes	*t*_1/2_ (min)	* CL_int, micr_ (µL/min/mg)	** CL_int_ (mL/min/kg)	*** CL_hep_ (mL/min/kg)
Mouse liver	1.18	1173.6	4594.6	11.95
Human liver	4.85	286.0	334.6	0.96

* *CL_int,micr_* (microsomal intrinsic clearance) = *ln2*/*t*_1/2_ × [volume of incubation medium (μL)/microsomal protein in incubation (mg)]; ** *CL_int_* (intrinsic clearance) = *CL_int,micr_* × (mg microsomes/g liver) × [liver mass (g)/body mass (kg)]; scaling factors according to Słoczyńska et al. (2019) [27] 45 mg of microsomal protein per g mouse and human liver tissue, 87 g and 26 g liver tissue per kg mouse and human body weight, respectively; *** CL_hep_: predicted hepatic clearance by the “well-stirred model”(see below).

**Table 4:** Metabolites of SH045 detected after incubation with liver microsomes from mouse (MLM) and human (HLM) and in mouse urine after administration of 20 mg/kg BW (i.v.).

Metabolite	Occurrence	MRM Transition ^a^	∆mass ^b^	Biotransformation	R_t_ (min) ^c^	Gradient Elution ^d^
M1a	MLM	348.3/136.1	−16	uncertain	0.64	5–90%
M1b	MLM, HLM				0.72	
M1c	MLM, HLM	348.3/256.1			4.13	
M2a	MLM, HLM	368.3/293.2	4	2-fold reduction (2x + 2)	3.14	20–90%
M2b	MLM, HLM				3.22	
M3a	Urine	380.3/305.3	16	hydroxylation	2.89	5–90%
M3b	Urine				3.04	
M3c	Urine				3.15	
M3d	Urine				3.26	
M3e	Urine				3.8	
M3f	MLM, HLM, Urine				3.88	
M3g	MLM, HLM, Urine				3.95	
M3h	MLM, HLM				3.96	
M3i	MLM, HLM, Urine				4.08	
M3j	MLM, HLM				4.24	
M3k	MLM, HLM				4.29	
M3l	MLM, HLM				4.72	
M3m	MLM, HLM				4.95	
M3n	MLM, HLM				5.08	
M3o	MLM, HLM				5.16	
M3p	MLM, HLM				5.25	
M3q	MLM, HLM				5.56	
M4a	Urine	382.3/197.2	18	reduction + hydroxylation (+2 + 16)	4.07	5–90%
M5a	MLM, HLM	396.3/352.2	32	hydroxylation + hydroxylation or epoxidation (2x + 16)	2.5	20–90%
M5b	MLM, HLM				2.57	
M6a	MLM, HLM	398.3/323.3	34	reduction + 2-fold hydroxylation (+2 + 2× 16)	3.15	20–90%
M6b	MLM, HLM				3.23	
M6c	MLM, HLM				0.63	85–90%
M6d	MLM, HLM	398.3/357.1			0.86	
M6e	Urine				5.72	
M7a	MLM, HLM, Urine	414.3/339.2	50	reduction + 3-fold hydroxylation (+2 + 3× 16)	2.73	5–90%
M7b	MLM, HLM, Urine				2.87	
M7c	MLM, HLM, Urine				2.99	
M7d	MLM, Urine				3.1	
M7e	MLM, Urine				3.53	
M7f	MLM				3.62	
M7g	MLM				3.76	
M7h	Urine	414.3/271.1			5.99	20–90%
M8a	MLM, HLM	540.3/346.3	176	glucuronidation	3.46	20–90%
M9a	HLM	556.3/539.4	192	hydroxylation + glucuronidation (+16 + 176)	0.64	70–90%
M9b	HLM				0.77	
M9c	MLM, Urine				4.91	
M10a	MLM, HLM	574.3/398.3	210	reduction + 2-fold hydroxylation + glucuronidation (+2 + 2× 16 + 176)	1.89	70–90%
M10b					3.88	

^a^ MRM transition: *m*/*z* of [M+H]+ to *m*/*z* of main fragment, ^b^ ∆mass: mass difference to SH045 ([M+H]+: *m*/*z* 364.3), ^c^ retention time, relates to the respective gradient elution system given in d, ^d^ gradient elution system for chromatographic separation: 5–90%, 20–90%, 85–90% or 70–90% ACN (0.1 % formic acid) in 0–6 min (see Supplementary Materials SM7); Rt of SH045 was 5.73, 5.23, 1.77 and 0.91 min, respectively.

## Data Availability

The data presented in this study are available on request from the corresponding authors.

## Supplementary Materials

Supporting information can be downloaded at: https://www.mdpi.com/article/10.3390/ijms23073635/s1 [17,19,22,27,28,30,33,43,44,45,46,47,48,49,50,51,52,53].
