# Supplementary material for: A Pharmacokinetic and Metabolism Study of the TRPC6 Inhibitor SH045 in Mice by LC-MS/MS
Source: Int J Mol Sci. 2022 Mar 26;23(7):3635. doi: 10.3390/ijms23073635 (PMC8998618; doi:10.3390/ijms23073635)
Supplement: Supplementary file 1 [file ijms-23-03635-s001.zip › ijms-1634709-supplementary.pdf]

International Journal of Molecular Sciences

MDPI

Supplementary Material

A Pharmacokinetic and Metabolism Study of the TRPC6 Inhibitor SH045 in Mice by LC-MS/MS

Xiao-Ning Chai [1,†], Friedrich-Alexander Ludwig [2,†], Anne Müglitz [1], Yuanyuan Gong [1], Michael Schaefer [1], Ralf Regenthal [3,‡] and Ute Krügel [1,*,‡]

[1] Rudolf Boehm Institute for Pharmacology and Toxicology, Leipzig University, 04107 Leipzig, Germany; xiao-ning.chai@medizin.uni-leipzig.de (X.-N.C.); anne.mueglitz@medizin.uni-leipzig.de (A.M.); gong.yuanyuan@medizin.uni-leipzig.de (Y.G.); michael.schaefer@medizin.uni-leipzig.de (M.S.); ute.kruegel@medizin.uni-leipzig.de (U.K)
[2] Department of Neuroradiopharmaceuticals, Institute of Radiopharmaceutical Cancer Research, Helmholtz-Zentrum Dresden-Rossendorf, 04318 Leipzig, Germany; f.ludwig@hzdr.de (F.-A.L.)
[3] Clinical Pharmacology, Rudolf Boehm Institute for Pharmacology and Toxicology, Leipzig University, 04107, Leipzig, Germany; ralf.regenthal@medizin.uni-leipzig.de (R.R.)
* Correspondence: ute.kruegel@medizin.uni-leipzig.de
† These authors contributed equally to this work.
‡ These authors contributed equally to this work.

Table of content

Methods and Materials
- SM1 Materials
- SM2 Quantification of SH045 by HPLC and tandem mass spectrometry (LC-MS/MS)
- SM3 Histopathological examination of tissue repeatedly exposed to SH045
- SM4 Preparation of samples for analysis of SH045 and its metabolites by LC-MS/MS
- SM5 Tissue binding of SH045
- SM6 Metabolism studies in MLM and HLM and calculation of hepatic clearance
- SM7 Identification of metabolism pathways and metabolites of SH045 in MLM and HLM
- SM8 Algorithm of *in vitro-in vivo* extrapolation of hepatic clearance

Results
- SR1 LC-MS/MS methods; Table S1
- SR2 Tissue binding of SH045; Table S2
- SR3 Histopathological investigations, Figure S1
- SR4 Parameters and resulting values for *in vitro-in vivo* extrapolation of hepatic clearance; Table S3
- SR5 Identification of metabolic pathways of SH045 and structural elucidation of metabolites
- Discussion of MRM chromatograms and EPI spectra of detected metabolites; Figures S2, S3, S4

References

Methods and Materials

SM1 Materials

Larixyl-*N*-methylcarbamate ((*1S,4S,4aR,8aS*)-4-((*S*)-3-hydroxy-3-methylpent-4-en-1-yl)-4a,8,8-trimethyl-3-methylenedeca-hydro-naphthalen-1-yl methylcarbamate, SH045, TRPC6 inhibitor) was synthesized as described by Häfner et al. [1]. (+)-Larixol (internal standard, IS) was isolated from *Larix decidua* turpentine [2]. Kolliphor® EL was obtained from Caelo (Hilden, Germany). Acetonitrile (ACN), water, and formic acid (LC-MS grade each) were purchased from Fisher Scientific (Schwerte, Germany). Testosterone, 4-nitrophenol, β-nicotinamide adenine dinucleotide 2′-phosphate reduced tetrasodium salt hydrate (β-NADPH), uridine 5′-diphosphoglucuronic acid ammonium salt (UDPGA), pilocarpine, ticlopidine, quercetin, sulfaphenazole, fluvoxamine, quinidine, 4-methyl pyrazole, ketoconazole, alamethicin and Dulbecco's phosphate-buffered saline (DPBS, pH 7.4) were obtained from Merck (Darmstadt, Germany). Ciprofloxacin was purchased from Bayer AG (Leverkusen, Germany). Liver microsomes from mice (CD-1, male, MLM) and human (pooled: 50 donors, HLM) were obtained from Fisher Scientific (Gibco, protein concentration: 20 mg/mL respectively). Syringe filters (Phenex, regenerated cellulose, pore-size 0.2 µm) were purchased from Phenomenex (Aschaffenburg, Germany) and centrifugal filter units (30 kDa) from Merck Millipore (County Cork, Ireland).

SM2 Quantification of SH045 by HPLC and tandem mass spectrometric methods (LC-MS/MS)

In brief, chromatographic separation was achieved using a Zorbax Eclipse XDB-C8 column (100 × 3.0 mm, 3.5 µm 80 Å, Agilent Technologies) with linear gradient elution using water and ACN, each with 0.1 % formic acid as additive, during a total run time of 20 min. For quantification (+)-larixol was used as IS. MS detection was achieved by multiple reaction monitoring (MRM) scan mode for SH045 (*m*/*z* 364.3/289.3) and IS (*m*/*z* 307.2/151.2), respectively. Further conditions and parameters were set as reported previously [3]. Freshly prepared calibration samples for SH045 in plasma and tissue comprised eight concentration levels analyzed in duplicates (2, 5, 10, 100, 200, 800, 1200 and 1600 ng/mL). For metabolism studies in microsomes, SH045 was dissolved in pure DMSO.

To identify metabolites of SH045 in microsomal fractions and urine chromatographic separation was achieved on a Poroshell 120 EC-C18 column (50 × 4.6 mm, 2.7 µm, Agilent Technologies) followed by linear gradient elution (% ACN in water, with 0.1 % formic acid in total): 0–6 min, 5, 20, 70 or 85–90%; 6–8 min, 90%; 8.1–10 min, 5, 20, 70 or 85%, at 25°C and a flow rate of 1 mL/min followed by a 0.5-min-wash. MS was operated at positive electrospray ionization mode as follows: temperature: 600°C; needle voltage: 5500 V; curtain gas, ion source gas I and ion source gas II: 35, 60 and 50 (arbitrary units), respectively. For first survey measurements of metabolites, MS parameters were based on optimized parameters for SH045 [3]. MRM and precursor ion scan methods were adapted to detect predicted metabolic modifications, such as hydroxylation (*m*/*z* + 16), oxygenation (*m*/*z* + 14), reduction (*m*/*z* +2), demethylation (*m*/*z* – 14), and glucuronidation (*m*/*z* + 176) as well as their multiple or combined occurrence. Further survey scans were performed using LightSight software. Identity of metabolites detected was finally confirmed by recording enhanced product ion spectra (EPI, scan rate: 10,000 Da/s, scan time: 100 ms) and subsequent analysis and comparison of fragmentation patterns. In some cases, these were verified by means of MS3 scans recorded after previous optimization of excitation energy (AF2) (data not shown). All analyses for metabolite identification included comparative measurements of appropriate control samples in order to avoid false-positive results.

SM3 Histopathological examination of tissue repeatedly exposed to SH045

Liver, kidney and lung were collected after repeated administration for inspection for histopathological changes, fixed in 4% paraformaldehyde solution and embedded in paraffin. Tissue slices (6 µm) of three animals either of untreated controls or such assigned to repeated administration of SH045 were deparaffinized and hematoxylin and eosin-stained. At least 5 slices per animal and tissue were microscopically examined using a light microscope (Zeiss, Germany) [4].

SM4 Preparation of samples for analysis of SH045 and its metabolites by LC-MS/MS

For tissue analysis, homogenates (0.1 g/mL in phosphate-buffered saline, PBS) were prepared with an ultrasonic homogenizer (Bandelin Sonopuls HD 2070, Bandelin electronic, Berlin, Germany) at 75% power in ice for 10 sec twice interrupted by 15 sec cooling down in between. 20 µL of homogenate or thawed plasma was mixed with 2 µL of IS (50 µg/mL), vortexed for 10 s, added with 178 µL ice-cold ACN in case of plasma and ACN/water (80:20) for tissue

homogenates, and shaken vigorously for 5 min. 40 µL urine was mixed with 4 µL of IS and extracted with 156 µL pure ACN.

All microsome incubations were stopped with 1 mL ice-cold ACN containing 5 µg/mL IS, vortexed for 1 min and cooled at -20 °C for 10 min.

After centrifugation of mixtures from tissue or microsomal incubations for 15 min at 4 °C, the supernatants were filtered through a 0.2-µm-pore-size syringe filter and stored in the autosampler at 6 °C until analysis by LC-MS/MS as mentioned above.

SM5 Tissue binding of SH045

The tissue binding of SH045 was analyzed exemplarily in lung, liver and kidney homogenate using ultrafiltration [5]. Appropriate concentrations of homogenate and SH045 were optimized out of data from a series of various dilution ratios of tissue homogenates (1/2, 1/5, 1/10 and 1/20, w/v) and SH045 concentrations in spiked homogenate (liver and kidney: 0.05, 0.1, 0.5, 1, 2, 5 µg/mL; lung: 1, 2, 5, 10, 20, 50 µg/mL, not shown). Tissue binding assays were performed as follows: 448 µL tissue homogenates (1/10, w/v) were mixed with 2 µL SH045 (final: 50 µg/ml for lung, 2 µg/ml for liver and 1 µg/ml kidney). After incubation for 15 min at 37 °C at 450 rpm, 400 µL of the mixture was transferred to centrifugal filter units (30kDa) and centrifuged for 20 min at 2000g and 37 °C. 20 µL of the mixture and the filtrate were analyzed for SH045. The concentration in the filtrate refers to the unbound SH045 concentration according to $tissue\ binding\ (\%) = (c_{tot} - c_{filtrate})\ /\ c_{total} \times 100\%$.

SM6 Metabolization studies in MLM and HLM and calculation of hepatic clearance

For determination of v_{max} and K_m of the NADPH-dependent microsomal depletion of SH045, the following incubations were performed for MLM and HLM, respectively. 2 µL SH045 (final concentration 0.5 – 100 µM, in triplicate), 12.5 µL HLM or MLM (finally 0.5 mg/mL protein) and 460.5 µL DPBS were vortexed and preincubated 37 °C for 5 min. The reaction was initiated by addition of 25 µL analogously pre-incubated β-NADPH (finally: 1 mM). After gentle shaking at 37 °C and 300 rpm for 10 min, incubations were terminated and samples prepared as described above.

Testosterone (10 µM in ACN/DPBS 2/3, v/v) instead of SH045 was used as positive control and analyzed by an appropriate HPLC method with UV detection.

Negative controls contained either no NADPH, no MLM or HLM or none of them. The overall velocity (v) of degradation was calculated from altered SH045 concentration (Δc) obtained from reduction peak area ratio of SH045 as: $v = \Delta c\ /\ incubation\ time \times concentration\ of\ enzyme \times\ volume\ (nmol/\ min \times mg)$. Mean maximum rate v_{max} and Michaelis-Menten constant K_m were calculated using Lineweaver-Burk transformation. From K_m an appropriate drug concentration of 2 µM was derived as prerequisite for experimental in vitro determination of $t_{1/2}$ required for further exploration of clearance.

For this purpose, microsomes were incubated as described above with 2 µM SH045 for indicated time intervals up to 30 min. The remaining concentrations of SH045 were determined to calculate the intrinsic microsomal clearance $CL_{int,micr}$ the intrinsic clearance CL_{int}, equal to the maximum enzyme activity of liver and for *in vitro-in vivo* exploration of hepatic clearance CL_{hep} in mouse and human using the "well-stirred" model [6].

SM7 Identification of metabolism pathways and metabolites of SH045 in MLM and HLM

To determine possible metabolism pathways of SH045 in MLM and HLM, an *in vitro* inhibition approach was performed with CYP-isoform specific inhibitors (final concentration): ciprofloxacin (CYP1A2; 150 µM), pilocarpine (CYP2A6; 50 µM), ticlopidine (CYP2B6; 5 µM), quercetin (CYP2C8; 2 µM), sulfaphenazole (CYP2C9; 10 µM), fluvoxamine (CYP2C19; 10 µM), quinidine (CYP2D6; 10 µM), 4-methyl pyrazole (CYP2E1; 100 µM) and ketoconazole (CYP3A4; 1 µM) [7, 8]. The inhibitors were dissolved in DPBS and appropriate methanol, the organic solvents not exceeding 1% (v/v).

SH045 (2 µM) was pre-incubated with MLM or HLM (0.5 mg/mL) and the specific inhibitor for 5 min at 37 °C in DPBS. Ticlopidine, a time-dependent inhibitor was pre-incubated for 15 min [9]. Incubations were performed in triplicate with NADPH (1 mM) in a final volume of 500 µL for 2 min and 10 min with pre-incubated MLM and HLM, respectively, terminated with 1 mL ice-cold ACN and processed as described above for LC-MS/MS. A sample without any inhibitor served as a positive control. Incubations with an equal volume of DMSO instead of SH045 and samples without NADPH, MLM or HLM or both, respectively, served as negative controls.

To explore metabolites of SH045 formed by microsomes, DPBS, alamethicin (25 µg/mL) [10] and MLM or HLM (1.0 mg/mL) were mixed and kept on ice for 15 min [11, 12]. SH045 (50 µM) and $MgCl_2$ (2 mM) were added, mixed and pre-incubated at 37 °C for 5 min. After addition of similarly pre-incubated NADPH (2 mM) and UDPGA (5 mM) to a final volume of 250 µL, mixtures were shaken gently at 37 °C for 120 min. Incubations were terminated and processed for LC-MS/MS analysis as described above. Incubations without NADPH and /or UDPGA, MLM or HLM, and SH045, respectively, served as negative controls as well as to provide conditions either for oxidation or glucuronidation.
Positive controls with testosterone (for oxidation) and 4-nitrophenol (for glucuronidation) instead of SH045 were analyzed using an appropriate HPLC method with UV detection to confirm complete conversion of both.

SM8 Algorithm of *in vitro-in vivo* extrapolation of hepatic clearance

For each used concentration (c_0) of SH045, the velocity (v) of enzymatic conversion was calculated from decrease of SH045 concentration ($c_0 - c_t$), during a reaction time (t) from "zero" to 10 min as follows:

$$v\ (nmol \times min^{-1} \times mg^{-1}) = (c_0\ (\mu M) - c_t\ (\mu M)) \times v_{inc}\ (mL) / t\ (min) / m_{protein}\ (mg)$$

v_{inc} is the total volume of the incubation mixture, $m_{protein}$ is the amount of protein of HLM or MLM in each incubation mixture of 0.5 mL and 0.25 mg used, respectively.
On the basis of the Michaelis-Menten equation the reciprocal velocity ($1/v$) was plotted *versus* the respective reciprocal concentration ($1/c_0$) of SH045 in a Lineweaver-Burk plot and v_{max} and K_m were determined by means of the following equation:

$$1 / v\ (nmol \times min^{-1} \times mg^{-1}) = K_m\ (\mu M) / v_{max}\ (nmol \times min^{-1} \times mg^{-1}) \times 1 / c_o\ (\mu M) + 1 / v_{max}\ (nmol \times min^{-1} \times mg^{-1})$$

Further clearance studies were performed with a deduced appropriate substrate concentration of SH045 of 2 µM. The remaining SH045 in microsomes at each time point t was calculated as:

$$Remaining\ percentage\ of\ SH045\ (\%) = (c_t / c_0) \times 100\%$$

The natural logarithm of remaining percentages of SH045 was plotted versus incubation time to obtain the slope ($-k$) from linear regression which allowed the calculation of in vitro half-life ($t_{1/2}$):

$$ln(Remaining\ percentage\ of\ SH045) = ln100 - k \times t \qquad t_{1/2} = ln2 / k$$

The intrinsic microsomal clearance ($CL_{int,micr}$) was calculated for mouse and human fractions:

$$CL_{int,micr}\ (\mu L \times min^{-1} \times kg^{-1}) = ln2 / t_{1/2}\ (min) \times v_{inc}\ (\mu L) / m_{protein}\ (mg)$$

$CL_{int,micr}$ is further used to estimate the *in vitro* intrinsic clearance (CL_{int}) of liver and to explore hepatic clearance (CL_{hep}) in vivo.

$$CL_{int}\ (mL \times min^{-1} \times kg^{-1}) = CL_{int,micr}\ (\mu L \times min^{-1} \times kg^{-1}) \times m_{liver}\ (g) / BW\ (kg) \times SF\ (mg/g)$$

The term m_{liver} (g)/BW (kg) refers to the mass of liver tissue per kilogram of body weight (BW), assumed to be 87 g/kg for mouse and 26 g/kg for human [12]. In the same manner, the scaling factor SF of 45 mg microsomal protein per gram of liver tissue was accepted for both species.
Finally, the hepatic clearance (CL_{hep}) for mouse and human could be estimated:

$$CL_{hep}\ (mL \times min^{-1} \times kg^{-1}) = Q\ (mL \times min^{-1} \times kg^{-1}) \times f_u \times CL_{int}\ (mL \times min^{-1} \times kg^{-1}) / Q + (f_u \times CL_{int}\ (mL \times min^{-1} \times kg^{-1}))$$

Q is the hepatic blood flow, assumed as 90 $mL \times min^{-1} \times kg^{-1}$ for mouse and 21 $mL \times min^{-1} \times kg^{-1}$ for human [13]. f_u is the unbound fraction of SH045 in plasma of 0.3% derived from the previously reported plasma protein binding of 99.7% for SH045 [3] .

Results

SR1 LC-MS/MS methods

Analytical quality parameters for matrix effect, extraction recovery and calibration curve in the various applications meet the requirements [3] and are summarized in Table S1. Besides quantification, LC-MS/MS was also required for detection and characterization of metabolites of SH045 in urine and microsomal preparations. For this

purpose, a time-saving chromatographic method was established allowing high throughput of measurements, necessary for variation of mass spectrometric parameters of different scan modes. Using a Poroshell 120 EC-C18 column (50 × 4.6 mm, 2.7 µm, Agilent Technologies) and elution with water/ ACN, containing 0.1% formic acid, at a flow rate of 1 mL/min, the overall runtime was 10 min and application of four different linear gradients enabled excellent separation of structurally similar metabolites (Table 4, respective chromatograms in Figure S3).

Table S1. Quality parameters for LC-MS/MS quantitative analysis of SH045 in various applications in mice (n = 3)

Tissues	Analyte	Extraction recovery (%)		Matrix effect (%)		Calibration curve		
		Mean (%)	RSD (%)	Mean (%)	RSD (%)	Range (ng/mL)	Equation	R^2
Liver	SH045 200 ng/mL	101.1	3.1	98.6	3.6	2 - 1600	y = 3.306x + -0.002	0.999
Kidney		100.0	1.9	103.0	2.5	2 - 1600	y = 3.025x + -0.001	0.999
Brain		98.9	8.2	104.1	1.3	2 - 1600	y = 7.875x + 0.003	0.999
Fat		93.8	1.0	100.0	2.0	2 - 1600	y = 4.259x + 0.006	0.999
Lung		97.2	1.8	99.7	2.1	2 - 1600	y = 3.704x + -0.005	0.999
Urine		105.9	0.1	100.0	0.1	2 - 500	y = 1.350x + 0.019	0.999

LC-MS/MS limit of detection and limit of quantification were 0.5 and 2 ng/mL, respectively.

SR2 Tissue binding of SH045

Table S2. Tissue homogenate binding of SH045 in kidney, liver, and lung (n = 3).

	Analyte	Tissues	30 min	60 min	120 min	240 min
Tissue binding (%)	SH045	Kidney	97.6 ± 0.9	97.8 ± 0.4	98.3 ± 0.2	98.3 ± 0.1
		Liver	99.4 ± 0.1	97.1 ± 1.0	99.0 ± 0.1	97.8 ± 0.9
		Lung	93.5 ± 1.2	94.9 ± 0.1	97.8 ± 0.5	97.4 ± 0.8

*The data are presented as the mean ± RSD. The concentration of SH045 in homogenates was: kidney (1 µg/mL), liver (2 µg/mL), lung (50 µg/mL) according to the c_{max} *in vivo*, respectively. The dilution ratio of tissue homogenate was optimized as 1:10 (w/v, g/mL) with PBS.

SR3 Histopathological investigations

To evaluate whether repeated administration of SH045 (20 mg/kg BW; i.v.) is toxicologically tolerable, we searched for histopathological alterations at hematoxylin-eosin stained tissues slices of mice liver, kidney and lung. Histological samples obtained from treated mice presented normal, without necrosis, edema, hemorrhage or infiltrations. Liver slices appeared normal in lobule structure with central vein, hepatocytes and sinusoids (Figure 3a). Similarly, the kidney tissue presents with intact renal corpuscle structure, including glomerulus and Bowman's capsule and proximal and distal tubules in the renal cortex (Figure 3b). In lung alveoli and inter-alveolar septa were of regular structure. No signs of septum thickening (Figure 3c). Further, no inflammation, infiltrates around bronchioles and pulmonary veins nor fibrosis were observed. Preparations with lower magnification compared to respective control slides are provided in Figure S1. Taken together, in the used study design with the higher dose no histopathological changes were observed.

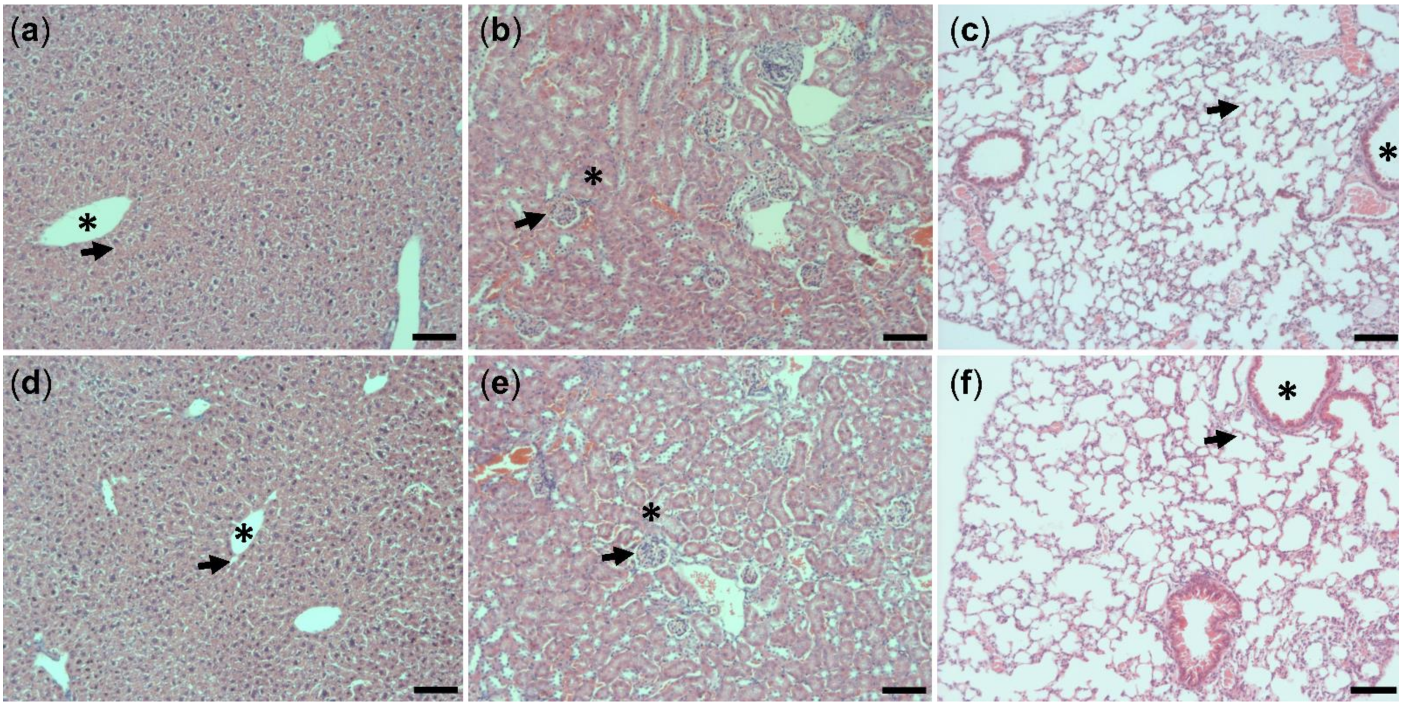

Figure S1. Hematoxylin-eosin overview stained tissues slices of mice liver (**a**,**d**), kidney (**b**,**e**) and lung (**c**,**f**) of vehicle treated controls (upper panel) and mice treated once a day for five days with SH045 (20 mg/kg BW, i.v.) (lower panel). asterisks: central vein (**a**,**d**), proximal tubule (**b**,**e**), bronchiole (**c**,**f**). Arrows: hepatic sinusoid (**a**,**d**), renal corpuscle (**b**,**e**), alveoli (**c**,**f**). Scale bar: 100 µm.

SR4 Parameters and resulting values for *in vitro-in vivo* extrapolation of hepatic clearance

Table S3. Values and scaling factors used and resultant intrinsic clearance parameters for *in vitro-in vivo* extrapolation of hepatic clearance of SH045.

Parameter	MLM	HLM
$t_{1/2}$	1.18	4.85
**Incubation volume (µL)**	500	500
**Microsomal protein (mg)**	0.25	0.25
$CL_{int,micr}$ **(µL/min/mg)**	1173	286
**Microsomes (mg) / liver (g)**	45	45
**Liver mass (g) / body mass (kg)**	26	87
CL_{int} **(mL/min/kg)**	4594	334
Q **(mL/min/kg)**	90	21
f_u	0.3	0.3
CL_{hep} **(mL/min/kg)**	11.95	0.96

SR 5 Identification of metabolic pathways of SH045 and structural elucidation of metabolites

To investigate whether metabolism is part of systemic clearance, experiments on cytochrome P450 isoenzyme inhibition were performed using isoform-specific inhibitors. It was shown that SH045 is mainly metabolized by the cytochrome P450 isoenzymes CYP3A4 and CYP2A6. For the majority of observed metabolic transformations hydroxylation appeared to be involved [14] (Figure 7; Table 4).

In detail, 17 metabolites (**M3a-q**) showed a mass shift of M+16, which corresponds to a possible mono-hydroxylation proven by fragmentation data of EPI spectra for most of them. However, for some of them with low

signal intensity, epoxidation at one of the carbon-carbon double bonds of SH045 could also be considered [15]. Moreover, sequential hydroxylations of SH045, of up to three times (**M7a-h**), occurred as well. Further, besides hydroxylation also reduction of carbon-carbon double bonds was involved in the formation of **M2a,b, M4a, M6a-e, M7a-h** and **M10a,b** [16].

Investigation of possible glucuronidation, one of the major phase II conjugation reactions [17], suggested the formation of the SH045-glucuronide **M8a** indicated by a characteristic mass shift of M+176. Further three metabolites (**M9a-c**) were assigned as glucuronides of previously formed hydroxylated metabolites of SH045, due to their mass shift of M+196. Analogously, EPI data suggested **M10a,b** to result from glucuronidation of **M6a,b** or M6c formed by previous reduction of one carbon-carbon double bond and di-hydroxylation. However, for some of the reported glucuronides detailed proof of identity was impaired by low signal intensity.

In urine, metabolites resulting from mono-hydroxylation of SH045 (**M3a-g, i**) were detected, too, of which five did not occur in liver microsomes. Metabolite **M9c**, which resulted from mono-hydroxylation and glucuronidation, was present in urine and samples from MLM incubations. The metabolites **M4a** and **M7h** appeared only in urine and may be assigned to reduction of one carbon-carbon double bond as well as to a single and a 3-fold hydroxylation of SH045, respectively. All detected metabolites and observed biotransformations are summarized in Table 4 and Figure 7. Relevant MRM chromatograms, EPI spectra, and interpretation of fragmentation patterns are given below.

Discussion of MRM chromatograms and EPI spectra of detected metabolites (Figures S2, S3, S4)

M2a, M2b (+4)

M2a and **M2b** were detected in MLM and HLM and showed a parent ion at *m*/*z* 368.3 $[M+4+H]^+$, respectively, which suggested a reduction of both carbon-carbon double bonds of the SH045 structure [16]. By reducing the exocyclic double bond an additional chiral center is formed bearing a methyl group is axial or equatorial position. This explains the detection of two epimeric metabolites showing very similar EPI spectra. Besides a fragment ion at *m*/*z* 293.2 resulting from loss of the N-methyl carbamate group (75 Da), fragment ions at *m*/*z* 249.1 and 231.2 are explained by subsequent stepwise cleavage in the pentyl side chain.

M3a-M3q (+16)

By monitoring an MRM transition of *m*/*z* 380.3/305.3 for samples from liver microsomes, 12 metabolites (**M3f-q**) were detected in total, of which **M3f**, **M3g,** and **M3i** appeared dominant and **M3g** showed the highest peak intensity. This applied for samples from both MLM and HLM, as proportions of these main metabolites were comparable for both. In contrast, samples from urine revealed in a total of 8 metabolites (**M3a-g**, **i**), of which **M3a-e** was not detected in liver microsomes. The highest peak intensity was found for **M3d**.

As observed for SH045, EPI spectra showed a neutral loss of 75 Da as one of the first fragmentation processes due to the cleavage of the N-methyl carbamate moiety. Another neutral loss of 18 Da was due to the elimination of water, resulting from a hydroxyl group already present in the structure of SH045. Regardless of order of occurrence, both mentioned neutral losses led to a fragment ion at *m*/*z* 287.2 in the case of **M3f**, **M3g**, **M3i-M3l**, **M3n,** and **M3o**. Another neutral loss of 18 Da resulted in the fragment ion at *m*/*z* 269.2 and provided evidence for a second hydroxyl group present in **M3f-M3l**, **M3n**, **M3o**, and **M3d** and therefore for a hydroxylation of the molecule. Besides, the observed elimination of water (18 Da) revealed that the formed hydroxyl group was not bound in beta position to a tertiary carbon atom, and therefore excluded a hydroxylation at one of the methyl groups directly bound to the decaline ring system. Instead of *m*/*z* 269.2, the fragmentation pattern of **M3m** contained a peak at *m*/*z* 271.3, which is in accordance with the EPI spectrum of SH045. Based on that, **M3m** was tentatively identified as a product of hydroxylation at the methyl group of the N-methyl carbamate moiety [18].

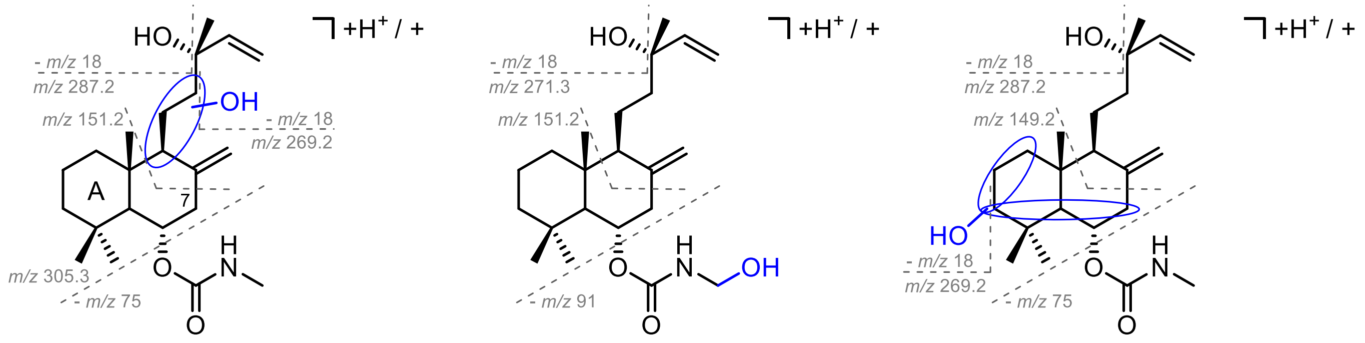

**M3f,g,i,k,o**	**M3m**	**M3b-c,e,j,l,p,q**
m/z 380.3	*m/z* 380.3	*m/z* 380.3
$[M+16+H]^+$	$[M+16+H]^+$	$[M+16+H]^+$

Figure S2. Fragmentation patterns observed during EPI scans and proposed structures of mono-hydroxylated metabolites of SH045 (mass shift +16).

However, for the other aforementioned metabolites, an elucidation of the site of hydroxylation in the molecule remains uncertain. Furthermore, for such metabolites for which 2-fold elimination of water was detected (**M3a,d**), epoxidation of one of the carbon-carbon double bonds instead of hydroxylation has to be considered [15].

According to previous interpretation of SH045's EPI data that an observed fragment ion at *m/z* 151.2 results from unchanged ring A of the labdane system, structures of some of the metabolites **M3a-M3q** might be assigned more precisely regarding their site of modification. Figure S2 illustrates a proposed interpretation of fragmentation patterns recorded.

M5a, M5b (+32)

For the metabolites **M5a** and **M5b**, both found only in MLM and HLM, the parent ion at *m/z* 396.3 $[M+32+H]^+$ can be explained by a dihydroxylation of SH045. A 2-fold elimination of water (18 Da) was observed in both EPI spectra, which appeared similar. However, another dehydration, as expected due to three hydroxyl groups in the molecule in total, was not visible directly in the EPI spectrum. Therefore it remains unclear whether the second introduction of oxygen occurred as a hydroxylation or epoxidation of one of the two carbon-carbon double bonds in the molecule [15]. However, since no cleavage of the N-methyl carbamate group (75 Da) was detected during fragmentation, a hydroxylation in particular at C-7 might be probable (Figure S4). This would explain e.g. the main fragment ion at *m/z* 276.0, which might result from dehydration, stabilizing the carbamate moiety, together with a cleavage in the pentenyl side chain.

M6a-M6e (+34)

M6a, **M6b**, and **M6c** could be separated chromatographically in samples from both MLM and HLM and showed similar fragmentation patterns. Besides the parent ion at m/z 398.3 $[M+34+H]^+$, the EPI spectra showed a base peak at *m/z* 323.2 due to cleavage of the N-methyl carbamate moiety (75 Da). Further, a 3-fold loss of water (18 Da) was observed. One resulted from the hydroxyl group present in SH045, and the second was highly likely resulting from hydroxylation. Since a third dehydration was observable, another hydroxylation was involved. Taking into account the overall mass shift of +34 for **M6a-e**, which might be a result of +16+16+2, an enzymatic reduction of one of the carbon-carbon double bonds can be concluded [16]. In contrast to **M6a-c**, **M6d** showed only one neutral loss of 18 Da (for explanation see M3a-q and M5a,b). However, from EPI data recorded for **M6e**, which was detected only in urine, a 3-fold neutral loss of *m/z* 18 could be concluded. However, the fragmentation pattern did not allow a clear interpretation as for **M6a-c**.

M7a-M7h (+50)

The parent ion at *m/z* 414.3 $[M+50+H]^+$ revealed 8 different metabolites (**M7a-h**) in total. Of them 7, 3, and 6 metabolites were detected in samples from MLM (**M7a-g**), HLM (**M7a-c**), and urine (**M7a-e, h**), respectively. Signals of **M7a** were of the highest intensity and the EPI spectra of **M7a** and **M7b** showed the significant neutral loss of 75 Da due to cleavage of the N-methyl carbamate group. Further, water (18 Da) was eliminated four times consecutively, which can be explained by presence of four hydroxyl groups as result of 3-fold hydroxylation. In addition, involvement of a

reduction of one carbon-carbon double bond as discussed for **M2a** and **M2b** as well as for **M6a-c** makes the mass difference of +50 plausible.

M8a (+176)

Metabolite **M8a**, detected only in MLM and HLM, showed a parent ion at *m*/*z* 540.4 $[M+176+H]^+$ as well as a fragment ion at *m*/*z* 364.3 resulting from characteristic cleavage of the glucuronic acid moiety (176 Da) [17].

M9a-M9c (+192)

Metabolites **M9a** and **M9b** were detected only after incubation of HLM in presence of both NADPH and UDPGA. In contrast, **M9c** was found only in MLM and mouse urine. For the more intense signal of **M9c**, the detected parent ion at *m*/*z* 556.3 $[M+16+176+H]^+$ clearly suggested an introduction of oxygen as well as glucuronidation, the latter further proven by a characteristic neutral loss of 176 Da [17]. However, although it appears highly likely that glucuronidation occurred subsequent to previous hydroxylation at a carbon atom, the EPI spectrum gave no evidence for this hypothesis.

M10a, M10b (+210)

For both metabolites, which were detected in microsomes only, the parent ion at *m*/*z* 574.3 $[M+2+16+16+176+H]^+$ indicated a reduction of one of the carbon-carbon double bonds, together with 2-fold hydroxylation and glucuronidation. The EPI spectrum of M10a showed a neutral loss of 176 Da, however, for **M10b** no clear interpretation was possible.

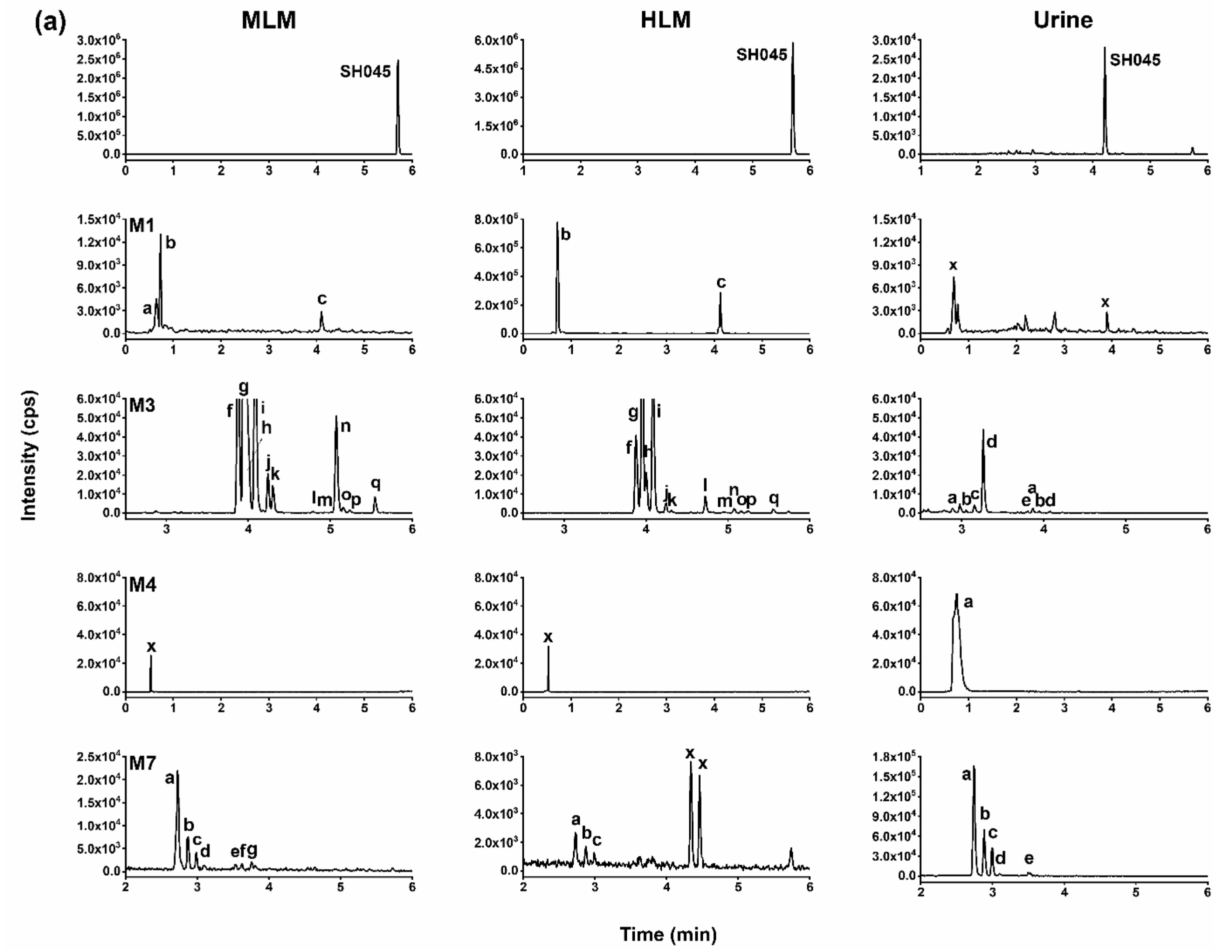

continued

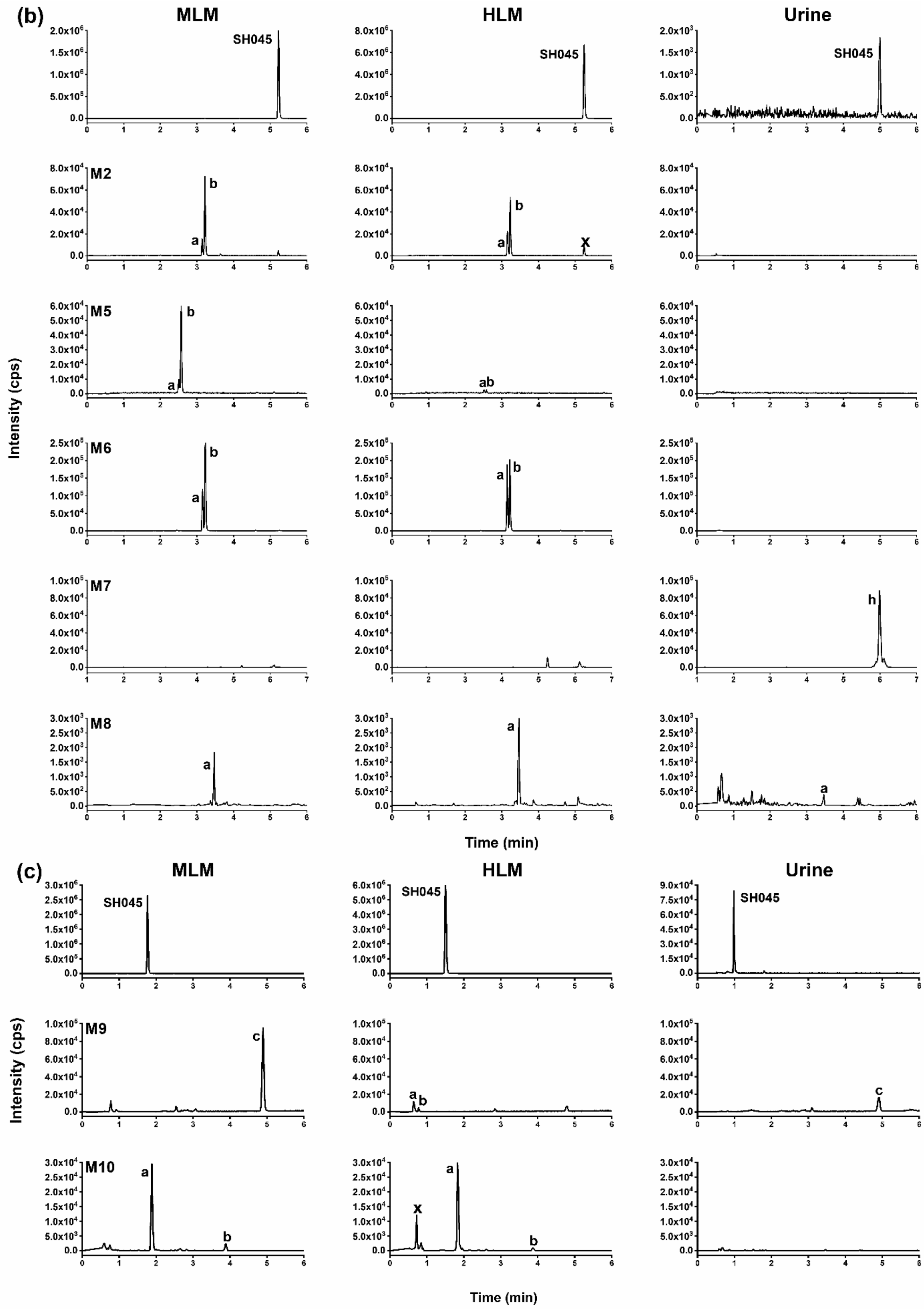
(b)
MLM
HLM
Urine
SH045
M2
M5
M6
M7
M8
Intensity (cps)
Time (min)
(c)
MLM
HLM
Urine
SH045
M9
M10
Intensity (cps)
Time (min)

continued

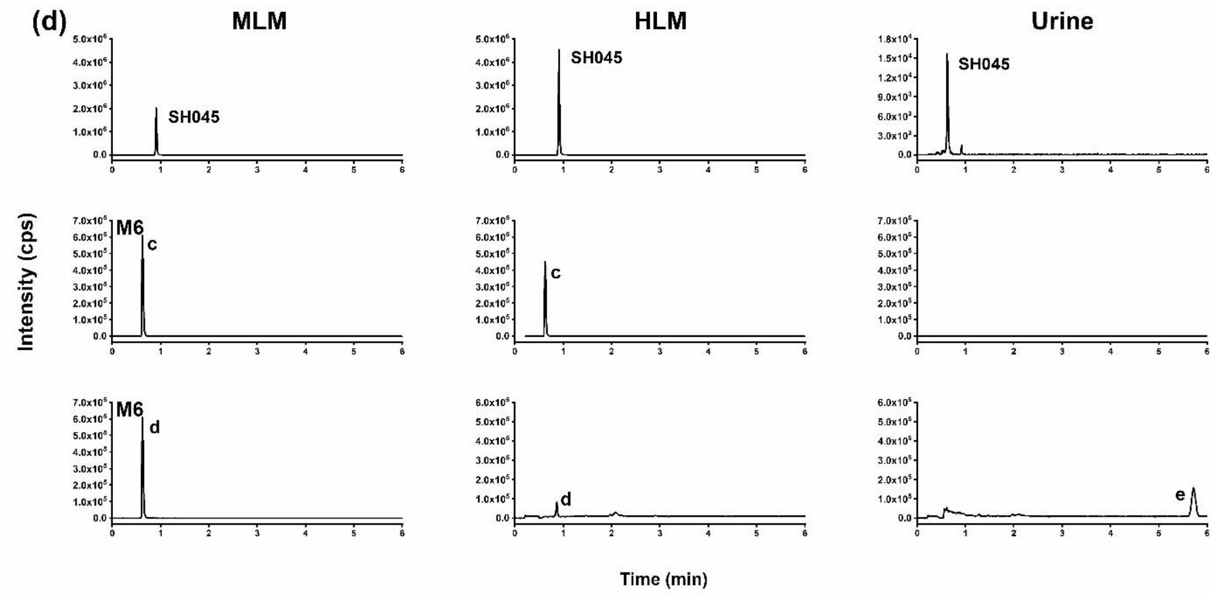

Figure S3. MRM chromatograms of detected metabolites of SH045 in mouse, human microsomes and urine after administration. MRM chromatograms of the metabolites and SH045 eluted in gradients of 5 – 90% ACN (**a**), 20 – 90% ACN (**b**), 70 – 90% ACN (**c**), 85 – 90% ACN (**d**) from 0 to 6 min. Peaks marked with "×" were also present in controls, either: without NADPH, without UDPGA, without both NADPH and UDPGA, without NADPH, UDPGA and liver microsomes or without SH045.

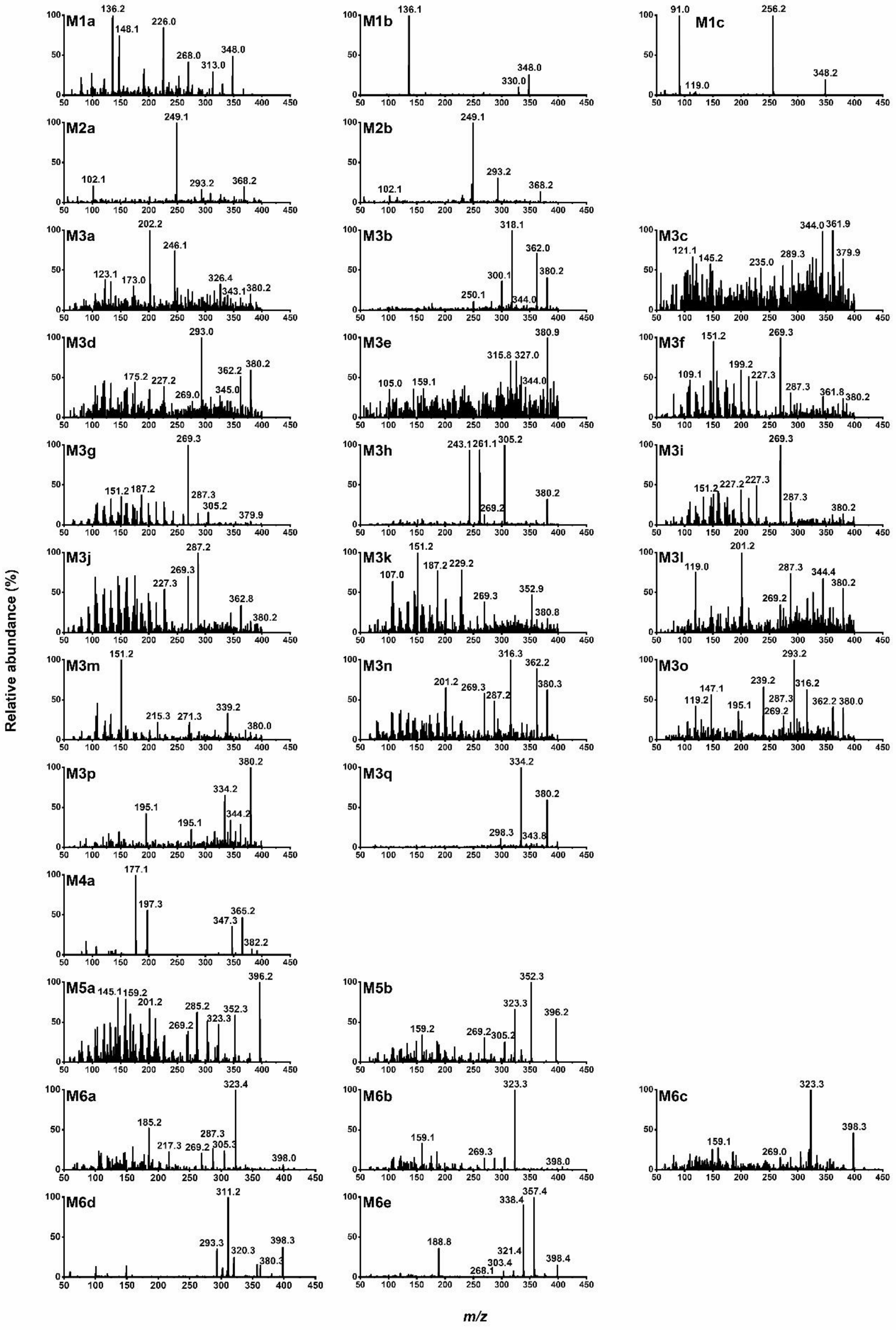
Relative abundance (%)
m/z
M1a 136.2 148.1 226.0 268.0 313.0 348.0
M1b 136.1 330.0 348.0
M1c 91.0 119.0 256.2 348.2
M2a 249.1 102.1 293.2 368.2
M2b 249.1 102.1 293.2 368.2
M3a 202.2 246.1 123.1 173.0 326.4 343.1 380.2
M3b 318.1 362.0 300.1 380.2 250.1 344.0
M3c 344.0 361.9 121.1 145.2 235.0 289.3 379.9
M3d 293.0 175.2 227.2 269.0 362.2 380.2 345.0
M3e 380.9 315.8 327.0 105.0 159.1 344.0
M3f 151.2 269.3 109.1 199.2 227.3 287.3 361.8 380.2
M3g 269.3 151.2 187.2 287.3 305.2 379.9
M3h 243.1 261.1 305.2 269.2 380.2
M3i 269.3 151.2 227.2 227.3 287.3 380.2
M3j 287.2 269.3 227.3 362.8 380.2
M3k 151.2 107.0 187.2 229.2 269.3 352.9 380.8
M3l 201.2 119.0 287.3 344.4 380.2 269.2
M3m 151.2 215.3 271.3 339.2 380.0
M3n 316.3 362.2 201.2 269.3 287.2 380.3
M3o 293.2 239.2 316.2 147.1 119.2 195.1 287.3 269.2 362.2 380.0
M3p 380.2 334.2 195.1 195.1 344.2
M3q 334.2 380.2 298.3 343.8
M4a 177.1 197.3 365.2 347.3 382.2
M5a 396.2 145.1 159.2 201.2 285.2 269.2 323.3 352.3
M5b 352.3 323.3 396.2 159.2 269.2 305.2
M6a 323.4 185.2 287.3 217.3 269.2 305.3 398.0
M6b 323.3 159.1 269.3 398.0
M6c 323.3 398.3 159.1 269.0
M6d 311.2 293.3 320.3 398.3 380.3
M6e 357.4 338.4 188.8 321.4 303.4 268.1 398.4

continued

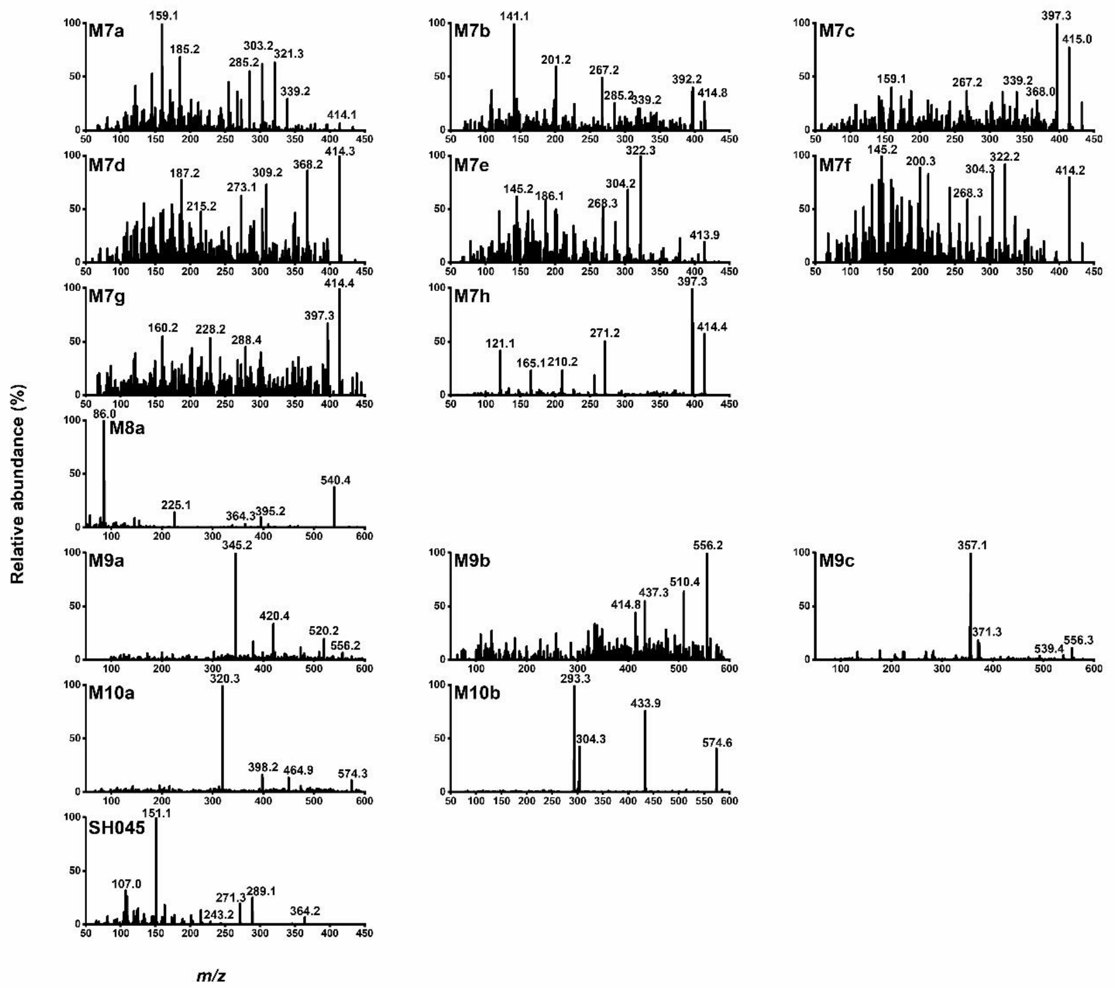

Figure S4. EPI spectra of detected SH045 metabolites in mouse and human microsomes and urine after administration of SH045 (20 mg/kg BW, i.v.).

References

1. Häfner, S.; Burg, F.; Kannler, M.; Urban, N.; Mayer, P.; Dietrich, A.; Trauner, D.; Broichhagen, J.; Schaefer, M. A (+)-Larixol Congener with High Affinity and Subtype Selectivity toward TRPC6. *ChemMedChem.* **2018**, 13, 1028–1035. DOI: 10.1002/cmdc.201800021. PMID: 29522264.
2. Urban, N.; Wang, L.; Kwiek, S.; Rademann, J.; Kuebler, W.M.; Schaefer, M. Identification and validation of larixyl acetate as a potent TRPC6 inhibitor. *Mol Pharmacol.* **2016**, 89, 197–213. DOI: 10.1124/mol.115.100792. PMID: 26500253.
3. Chai, X.-N.; Ludwig, F.-A.; Müglitz, A.; Schaefer, M.; Yin, H.-Y.; Brust, P.; Regenthal, R.; Krügel, U. Validation of an LC-MS/MS Method to Quantify the New TRPC6 Inhibitor SH045 (Larixyl *N*-methylcarbamate) and Its Application in an Exploratory Pharmacokinetic Study in Mice. *Pharmaceuticals* **2021**, 14, 259. DOI: 10.3390/ph14030259. PMID: 33805686.
4. Feldman AT, Wolfe D. Tissue processing and hematoxylin and eosin staining. *Methods Mol Biol.* **2014**, 1180, 31-43. DOI: 10.1007/978-1-4939-1050-2_3. PMID: 25015141.
5. Ayyar, V.S.; Song, D.; DuBois, D.C.; Almon, R.R.; Jusko, W.J. Modeling Corticosteroid Pharmacokinetics and Pharmacodynamics, Part I: Determination and Prediction of Dexamethasone and Methylprednisolone Tissue Binding in the Rat. *J Pharmacol Exp Ther.* **2019**, 370, 318–326. DOI: 10.1124/jpet.119.257519. PMID: 31197020.
6. Pang, K.S.; Han, Y.R.; Noh, K.; Lee, P.I.; Rowland, M. Hepatic clearance concepts and misconceptions: why the well-stirred model is still used even though it is not physiologic reality? *Biochem Pharmacol.* **2019**, 169, 113596. DOI: 10.1016/j.bcp.2019.07.025. PMID: 31398312.
7. Hakkola, J.; Hukkanen, J.; Turpeinen, M.; Pelkonen, O. Inhibition and induction of CYP enzymes in humans: an update. *Arch Toxico.* **2020**, 94, 3671–3722. DOI: 10.1007/s00204-020-02936-7. PMID: 33111191.

8. Food and Drug Administration. Drug Development and Drug Interactions: Table of Substrates, Inhibitors and Inducers, New York, USA, **2021**. https://www.fda.gov/drugs/drug-interactions-labeling/drug-development-and-drug-interactions-table-substrates-inhibitors-and-inducers
9. Talakad, J.C.; Shah, M.B.; Walker, G.S.; Xiang, C.; Halpert, J.R.; Dalvie, D. Comparison of in vitro metabolism of ticlopidine by human cytochrome P450 2B6 and rabbit cytochrome P450 2B4. *Drug Metab Dispos.* **2011**, 39, 539–550. DOI: 10.1124/dmd.110.037101. PMID: 21156812.
10. Fisher, M.B.; Campanale, K.; Ackermann, B.L.; VandenBranden, M.; Wrighton, S.A. In vitro glucuronidation using human liver microsomes and the pore-forming peptide alamethicin. *Drug Metab Dispos.* **2000**, 28, 560–566. PMID: 10772635.
11. Ludwig, F.-A.; Fischer, S.; Houska, R.; Hoepping, A.; Deuther-Conrad, W.; Schepmann, D.; Patt, M.; Meyer, P.M.; Hesse, S.; Becker, G.-A. In vitro and in vivo Human Metabolism of (S)-[18F] Fluspidine–A Radioligand for Imaging σ1 Receptors With Positron Emission Tomography (PET). *Front Pharmacol.* **2019**, 10, 534. DOI: 10.3389/fphar.2019.00534. PMID: 31263411.
12. Słoczyńska, K.; Gunia-Krzyżak, A.; Koczurkiewicz, P.; Wójcik-Pszczoła, K.; Żelaszczyk, D.; Popiół, J.; Pękala, E. Metabolic stability and its role in the discovery of new chemical entities. *Acta Pharm.* **2019**, 69, 345–361. DOI: 10.2478/acph-2019-0024. PMID: 31259741.
13. Davies, B.; Morris, T. Physiological parameters in laboratory animals and humans. *Pharm Res.* **1993**, 10, 1093–1095. DOI: 10.1023/a:1018943613122. PMID: 8378254.
14. Testa, B.; Krämer, S.D. The biochemistry of drug metabolism–an introduction: Part 2. Redox reactions and their enzymes. *Chem Biodivers.* **2007**, 4, 257–405. DOI: 10.1002/cbdv.200790032. PMID: 17372942.
15. Visser, S.P. de; Ogliaro, F.; Sharma, P.K.; Shaik, S. What factors affect the regioselectivity of oxidation by cytochrome P450? A DFT study of allylic hydroxylation and double bond epoxidation in a model reaction. *J Am Chem Soc.* **2002,** 124, 11809–11826. DOI: 10.1021/ja026872d. PMID: 12296749.
16. Huang, M.; Hu, H.; Ma, L.; Zhou, Q.; Yu, L.; Zeng, S. Carbon–carbon double-bond reductases in nature. *Drug Metab Rev.* **2014**, 46, 362–378. DOI: 10.3109/03602532.2014.910219. PMID: 24750117.
17. Levsen, K.; Schiebel, H.-M.; Behnke, B.; Dötzer, R.; Dreher, W.; Elend, M.; Thiele, H. Structure elucidation of phase II metabolites by tandem mass spectrometry: an overview. *J Chromatogr A.* **2005**, 1067, 55–72. DOI: 10.1016/j.chroma.2004.08.165. PMID: 15844510.
18. Hassan, A.; Zayed, S.; Abdel-Hamid, F.M. Metabolism of carbamate drugs—I: Metabolism of 1-naphthyl-N-methyl carbamate (Sevin) in the rat. *Biochem. Pharmacol.* **1966**, 15, 2045–2055. DOI: 10.1016/0006-2952(66)90233-4. PMID: 5973174.
